# Precision and Accuracy in Quantitative Measurement of Gene Expression from Single-cell/nucleus RNA Sequencing Data

**DOI:** 10.1093/gpbjnl/qzaf077

**Published:** 2025-08-26

**Authors:** Rujia Dai, Ming Zhang, Tianyao Chu, Richard Kopp, Chunling Zhang, Kefu Liu, Yue Wang, Xusheng Wang, Chao Chen, Chunyu Liu

**Affiliations:** Department of Psychiatry, SUNY Upstate Medical University, Syracuse, NY 13210, USA; MOE Key Laboratory of Rare Pediatric Diseases & Hunan Key Laboratory of Medical Genetics, School of Life Sciences, and Department of Psychiatry, The Second Xiangya Hospital, Central South University, Changsha 410205, China; MOE Key Laboratory of Rare Pediatric Diseases & Hunan Key Laboratory of Medical Genetics, School of Life Sciences, and Department of Psychiatry, The Second Xiangya Hospital, Central South University, Changsha 410205, China; Department of Psychiatry, SUNY Upstate Medical University, Syracuse, NY 13210, USA; Department of Neuroscience & Physiology, SUNY Upstate Medical University, Syracuse, NY 13210, USA; MOE Key Laboratory of Rare Pediatric Diseases & Hunan Key Laboratory of Medical Genetics, School of Life Sciences, and Department of Psychiatry, The Second Xiangya Hospital, Central South University, Changsha 410205, China; Department of Electrical and Computer Engineering, Virginia Polytechnic Institute and State University, Virginia, VA 24061, USA; Department of Neurology, University of Tennessee Health Science Center, Memphis, TN 38163, USA; Department of Genetics, Genomics, and Informatics, University of Tennessee Health Science Center, Memphis, TN 38163, USA; MOE Key Laboratory of Rare Pediatric Diseases & Hunan Key Laboratory of Medical Genetics, School of Life Sciences, and Department of Psychiatry, The Second Xiangya Hospital, Central South University, Changsha 410205, China; Furong Laboratory, Changsha 410008, China; Hunan Key Laboratory of Animal Models for Human Diseases, Central South University, Changsha 410008, China; Department of Psychiatry, SUNY Upstate Medical University, Syracuse, NY 13210, USA; MOE Key Laboratory of Rare Pediatric Diseases & Hunan Key Laboratory of Medical Genetics, School of Life Sciences, and Department of Psychiatry, The Second Xiangya Hospital, Central South University, Changsha 410205, China; Department of Neuroscience & Physiology, SUNY Upstate Medical University, Syracuse, NY 13210, USA

**Keywords:** Single-cell RNA sequencing, Single-nucleus RNA sequencing, Single-cell genomics, Transcriptome, Quality control

## Abstract

Single-cell RNA sequencing (scRNA-seq) and single-nucleus RNA sequencing (snRNA-seq) have become essential tools for profiling gene expression across different cell types in biomedical research. While factors like RNA integrity, cell count, and sequencing depth are known to influence data quality, quantitative benchmarks and actionable guidelines are lacking. This gap contributes to variability in study designs and inconsistencies in downstream analyses. In this study, we systematically evaluated quantitative precision and accuracy in expression measures across 23 sc/snRNA-seq datasets comprising 3,682,576 cells from 339 samples. Precision was assessed using technical replicates based on pseudo-bulks created from subsampling. Accuracy was evaluated using sample-matched scRNA-seq and pooled-cell RNA sequencing data of mononuclear phagocytes from four species. Our results show that precision and accuracy are generally low at the single-cell level, with reproducibility being strongly influenced by cell count and RNA quality. We established data-driven thresholds for optimizing study design, recommending at least 500 cells per cell type per individual to achieve reliable quantification. Furthermore, we showed that signal-to-noise ratio is a key metric for identifying reproducible differentially expressed genes. To support future research, we developed Variability In single-Cell gene Expression (VICE), a tool that evaluates sc/snRNA-seq data quality and estimates the true positive rate of differential expression results based on sample size, observed noise levels, and expected effect size. These findings provide practical, evidence-based guidelines to enhance the reliability and reproducibility of sc/snRNA-seq studies.

## Graphical abstract

**Figure:**
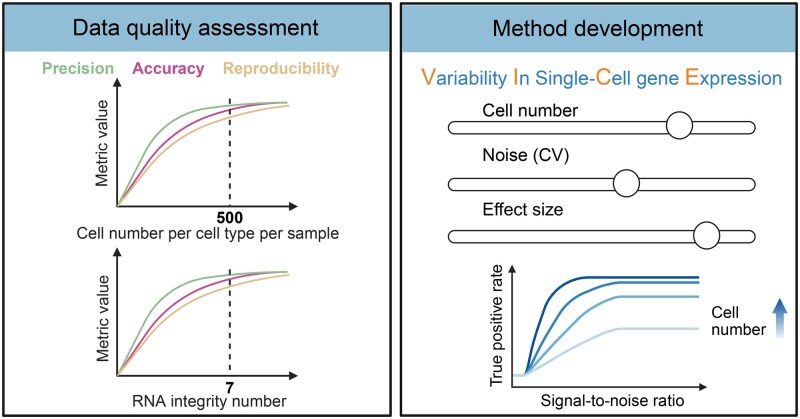

## Introduction

Single-cell RNA sequencing (scRNA-seq) and single-nucleus RNA sequencing (snRNA-seq) are powerful technologies developed for measuring gene expression in individual cells. The first scRNA-seq study was published in 2009 by Tang and his colleagues [1]. Smart-seq was developed, enabling the amplification and sequencing of full-length messenger RNA (mRNA) transcripts from individual cells, characterizing transcriptomes at single-cell resolution. Since then, more technologies have been developed for single-cell profiling [2], with 10X Chromium and Smart-seq being the two most commonly used methods.

scRNA-seq and snRNA-seq have been used in various applications, including identifying novel transcriptional regulatory mechanisms [3], characterizing cell types and tissue compositions [4], studying developmental dynamics and trajectories of different cell types [5,6], and identifying cell-type-specific changes as biomarkers for disease or treatment responses [7–9]. All these studies rely on accurate and precise measures of gene expression in each cell type. Precision and accuracy in the quantitative measurement of gene expression are defined as the variability of expression across replicates and the degree to which expression measurements match the actual or true values, hereafter referred to as precision and accuracy, respectively. Only when gene expression is quantified precisely and accurately in each sample, can the results of downstream analyses be reproducible and meaningful.

Random and systematic technical variability adds noise to the expression measurements in sc/snRNA-seq [10]. Many zero values are observed in sc/snRNA-seq data, called “dropouts” [11]. Dropouts can be caused either by the true absence of target gene expression or by technical factors such as low mRNA input, capture efficiency, amplification efficiency, and sequencing depth. These technical factors can reduce precision and cause bias in accuracy of gene expression measurements. Previous studies have attempted to assess technical noise in scRNA-seq data using spike-ins [12], sample-matched bulk-tissue RNA sequencing (RNA-seq) data [13], or quantitative polymerase chain reaction (qPCR) [14] as references. However, these methods have rarely been used due to costs and practical limitations.

Strategies to improve the quality of single-cell data such as pooling more cells have been developed, but standardized procedures for completing sc/snRNA-seq are lacking. There is a lack of systematic, quantitative thresholds to guide experimental design, making it challenging to define optimal parameters for achieving reliable results. These factors are often inconsistently evaluated across published studies, resulting in variability in data quality assessment. Practical guidelines — such as the minimum number of cells required per cell type — are either lacking or too vague, leaving researchers without clear direction for ensuring robust data quality in their experiments.

**Figure 1:**
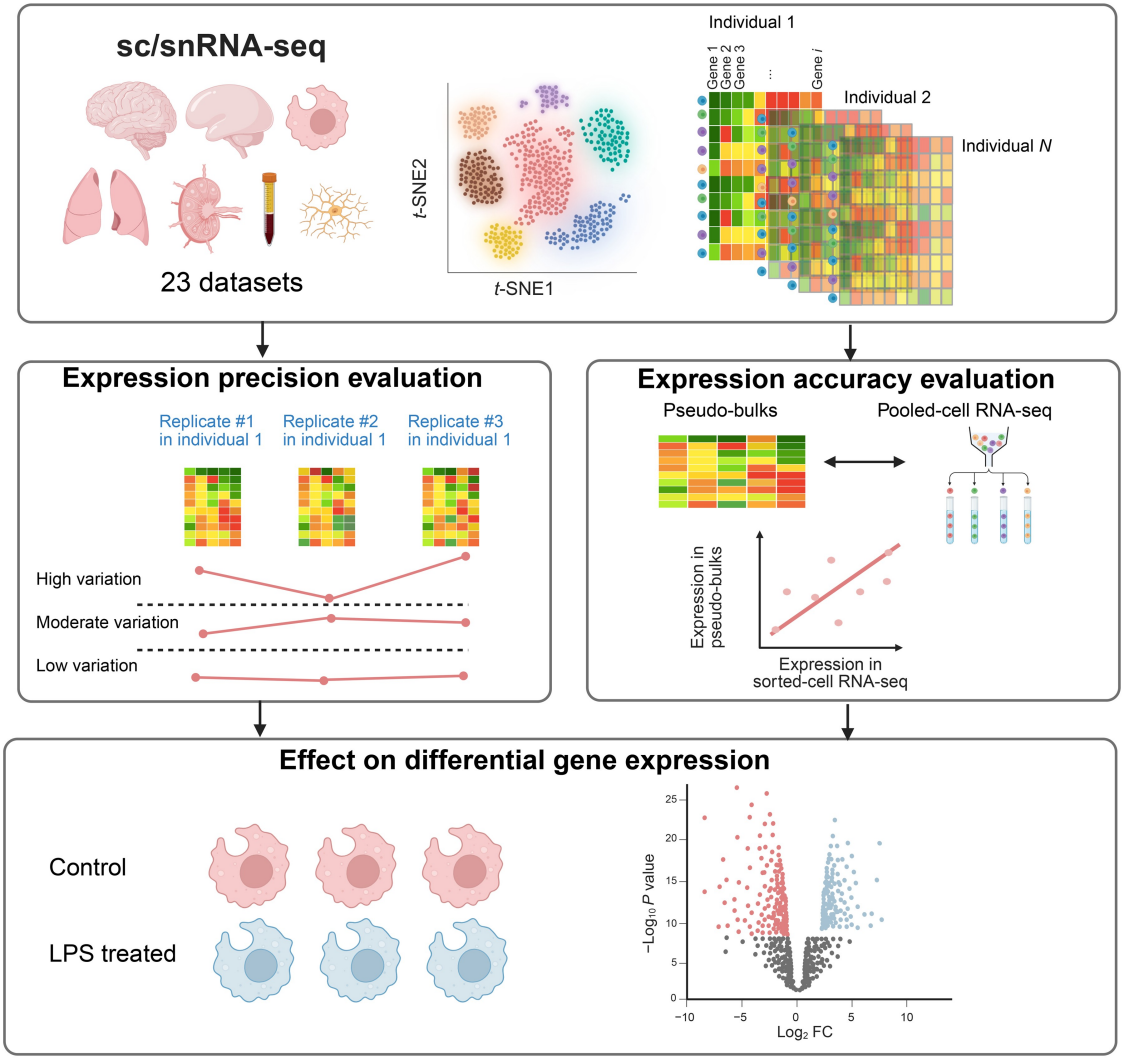
Overview of the study Framework for evaluating the expression precision and accuracy of sc/snRNA-seq across datasets and platforms. We assessed the precision and accuracy of gene expression measurements using 23 sc/snRNA-seq datasets generated on three platforms. These datasets, published in high-profile journals, were derived from large consortium efforts, including the BICCN, reflecting current technological standards. Our analysis began with a survey of cell numbers and missing rates across datasets, followed by the evaluation of precision based on technical replicates. We then examined the influence of technical factors such as RNA quality, sequencing saturation rate, total read count, and platform type on expression precision. To assess accuracy, we compared scRNA-seq data from four cultured mononuclear phagocyte datasets with corresponding pooled-cell RNA-seq data from the same samples. Finally, we analyzed the effects of cell numbers and other factors on the reproducibility of downstream DE analyses. This figure was created with BioRender.com. sc/snRNA-seq, single-cell/nucleus RNA sequencing; RNA-seq, RNA sequencing; DE, differential expression; BICCN, BRAIN Initiative Cell Census Network; LPS, lipopolysaccharide; FC, fold change; *t*-SNE, *t*-distributed stochastic neighbor embedding.

In this study, we evaluated the precision and accuracy of expression measurements using 23 sc/snRNA-seq datasets generated on three different platforms and published in high-impact journals (**Table 1**) within the framework as illustrated in **Figure 1**. Initially, we surveyed the cell numbers and missing rates in these sc/snRNA-seq data, followed by calculating precision in each dataset using technical replicates based on pseudo-bulks. Additionally, we explored the impact of several technical factors, including RNA quality, saturation rate, total read count, and sequencing platform, on expression precision. We also evaluated the expression accuracy with four datasets of cultured mononuclear phagocytes from sample-matched pooled-cell RNA-seq and scRNA-seq data. Lastly, we evaluated the effect of cell number and other factors on the reproducibility of downstream differential expression (DE) analysis. Based on the evaluation, we provided practical guidelines for future studies. To facilitate future experiment design and data evaluation, we developed a tool named Variability In single-Cell gene Expression (VICE) at https://github.com/RujiaDai/VICE.

**Table 1:** sc/snRNA-seq datasets assessed in this study

Data label	Species	PMID	Tissue	No. of samples	No. of cells	No. of genes	No. of cell types	Platform
ROSMAP	Human	31042697	Prefrontal cortex	24	75,060	17,926	8	10X
autism_PFC	Human	31097668	Prefrontal cortex	10	29,900	27,563	8	10X
autism_ACC	Human	31097668	Anterior cingulate cortex	16	22,065	27,072	8	10X
MTG	Human	31435019	Medial temporal gyrus	8	15,583	43,474	7	Smart-seq
M1_10X	Human	34616062	Primary motor cortex	2	76,533	27,933	8	10X
GSE97930_vc	Human	29227469	Visual cortex	3	19,368	21,273	8	Drop-seq
GSE97930_fro	Human	29227469	Frontal cortex	1	19,368	18,751	8	Drop-seq
GSE140231	Human	32826893	Cortex	5	10,581	24,702	6	10X
TREM	Human	31932797	Prefrontal cortex	11	36,671	36,601	6	10X
GSE174367	Human	34239132	Prefrontal cortex	8	21,996	25,392	6	10X
BICCN_adult	Human	37824663	Cortex	4	130,5075	19,762	30	10X
BICCN_HVS	Human	37824649	Cortex	78	353,194	18,797	24	10X
BICCN_trimester1	Human	37824650	Cortex	21	789,139	33,538	12	10X
BICCN_dev	Human	37824647	Cortex	106	709,372	19,005	9	10X
Tabula Sapiens	Human	35549404	Lung	3	33,222	23,739	3	10X
Tabula Sapiens	Human	35549404	Blood	6	37,892	21,147	1	10X
Tabula Sapiens	Human	35549404	Lymph node	3	47,891	22,271	1	10X
Thrupp et al. [18]	Human	32997994	Microglia	3	14,823	21,015	1	10X
Thrupp et al. [18]	Human	32997994	Microglia	3	3940	27,891	1	10X (whole cell)
Hagai et al. [43]*	Mouse	30356220	Mononuclear phagocyte	6	17,776	15,319	1	Smart-seq2
Hagai et al. [43]*	Rat	30356220	Mononuclear phagocyte	6	13,277	15,150	1	Smart-seq2
Hagai et al. [43]*	Rabbit	30356220	Mononuclear phagocyte	6	17,097	9263	1	Smart-seq2
Hagai et al. [43]*	Pig	30356220	Mononuclear phagocyte	6	12,753	8906	1	Smart-seq2

*Note*:

* means with sample-matched sorted-cell RNA-seq data. RNA-seq, RNA sequencing; scRNA-seq, single-cell RNA sequencing; snRNA-seq, single-nucleus RNA sequencing.

## Results

### Existing sc/snRNA-seq data have high missing rates

**Figure 2:**
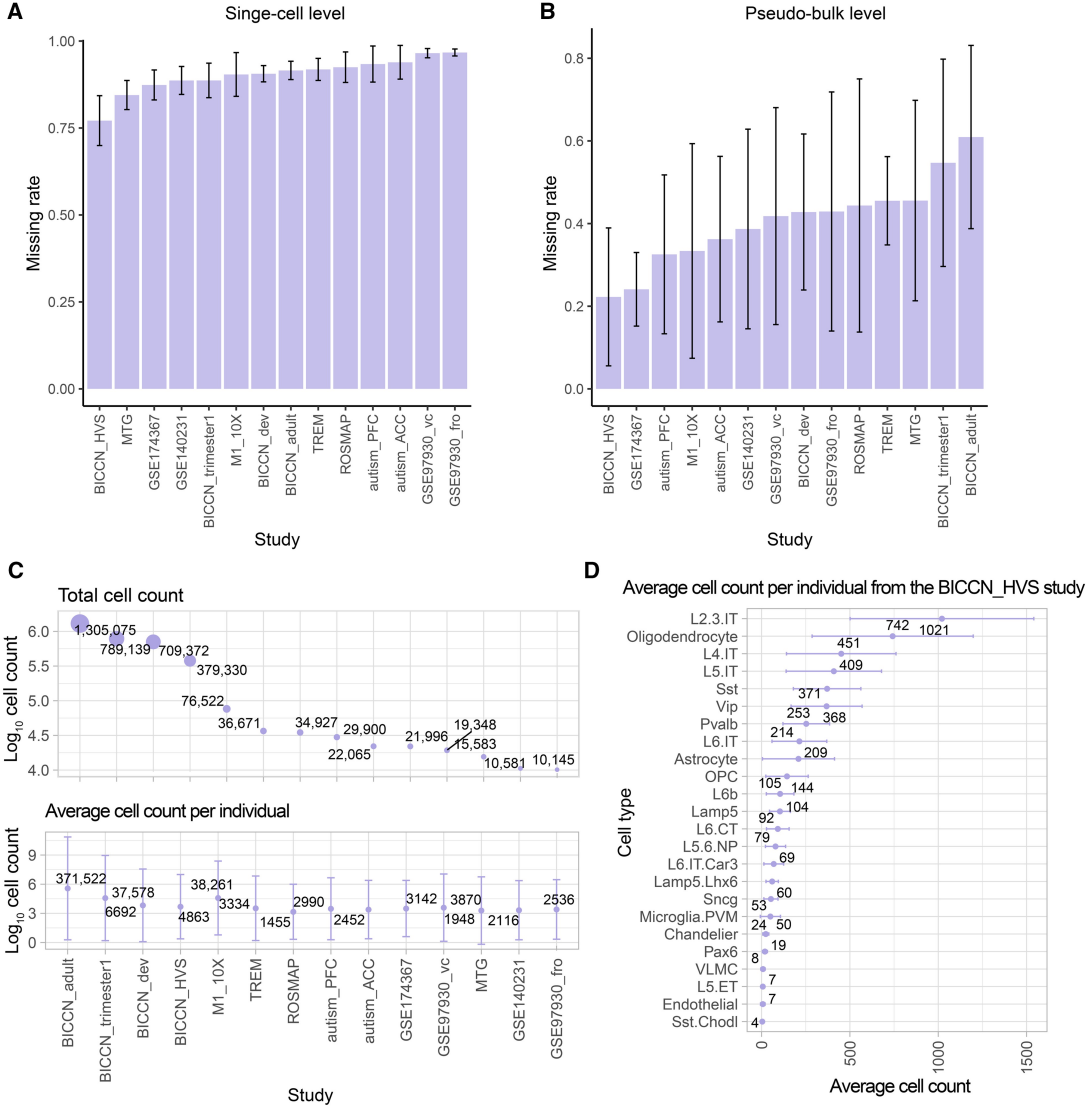
Missing rates and cell numbers in 14 datasets **A.** Missing rate per gene at single-cell level. **B.** Missing rate per gene at the pseudo-bulk level. **C.** Total count of cells studied, along with the average cell count per individual. The average cell count per individual was calculated by dividing the total cell count by the individual count in the study. Cell count was log_10_-transformed for better visualization. **D.** An illustrative example showcasing the average cell count per individual, specifically drawn from the BICCN_HVS study.

We measured the missing rate for each gene at both the single-cell and pseudo-bulk levels. Pseudo-bulks were created from single-cell gene expression of a specific cell type within an individual to mimic bulk RNA-seq data. The missing rate was defined as the proportion of cells with zero expression for a given gene across all single cells or pseudo-bulks of the same cell type. Single cells had an average missing rate of 90% (**Figure 2**A), while the pseudo-bulks reduced the average missing rate to 40% (Figure 2B). Including more cells in the pseudo-bulks resulted in a lower observed missing rate (Figure S1).

Though each project sequenced many cells, we noticed that the number of cells sequenced per cell type per individual was sometimes very small, particularly for minor cell types. Across the 14 brain datasets, the average total cell count was 247,190, whereas the average cell number per individual was 34,483 (Figure 2C). The number was even much smaller for specific cell types per individual. For instance, the BICCN_HVS study sequenced 353,194 cells and categorized 24 cell classes (Figure 2D). The largest group of cells in this dataset comprised an average of 1021 intratelencephalic (IT) neurons from layers 2 and 3, while the smallest group had only an average of 4 somatostatin (SST) chodl inhibitory neurons across the samples, a difference of three orders of magnitude.

### Low expression precision in sc/snRNA-seq data

Expression precision was evaluated by the expression variability across technical replicates based on pseudo-bulks in sc/snRNA-seq data. First, we generated technical replicates based on pseudo-bulks by randomly grouping cells of the same type from the same individual into three groups and totaling expression values of each gene from all cells within each group (Figure S2). We then calculated the coefficient of variation (CV) for each gene to measure the variability of gene expression across the technical replicates based on pseudo-bulks in each cell type. To avoid sampling bias, we calculated the CV 100 times and used the averaged CV to represent the overall precision in the data.

**Figure 3:**
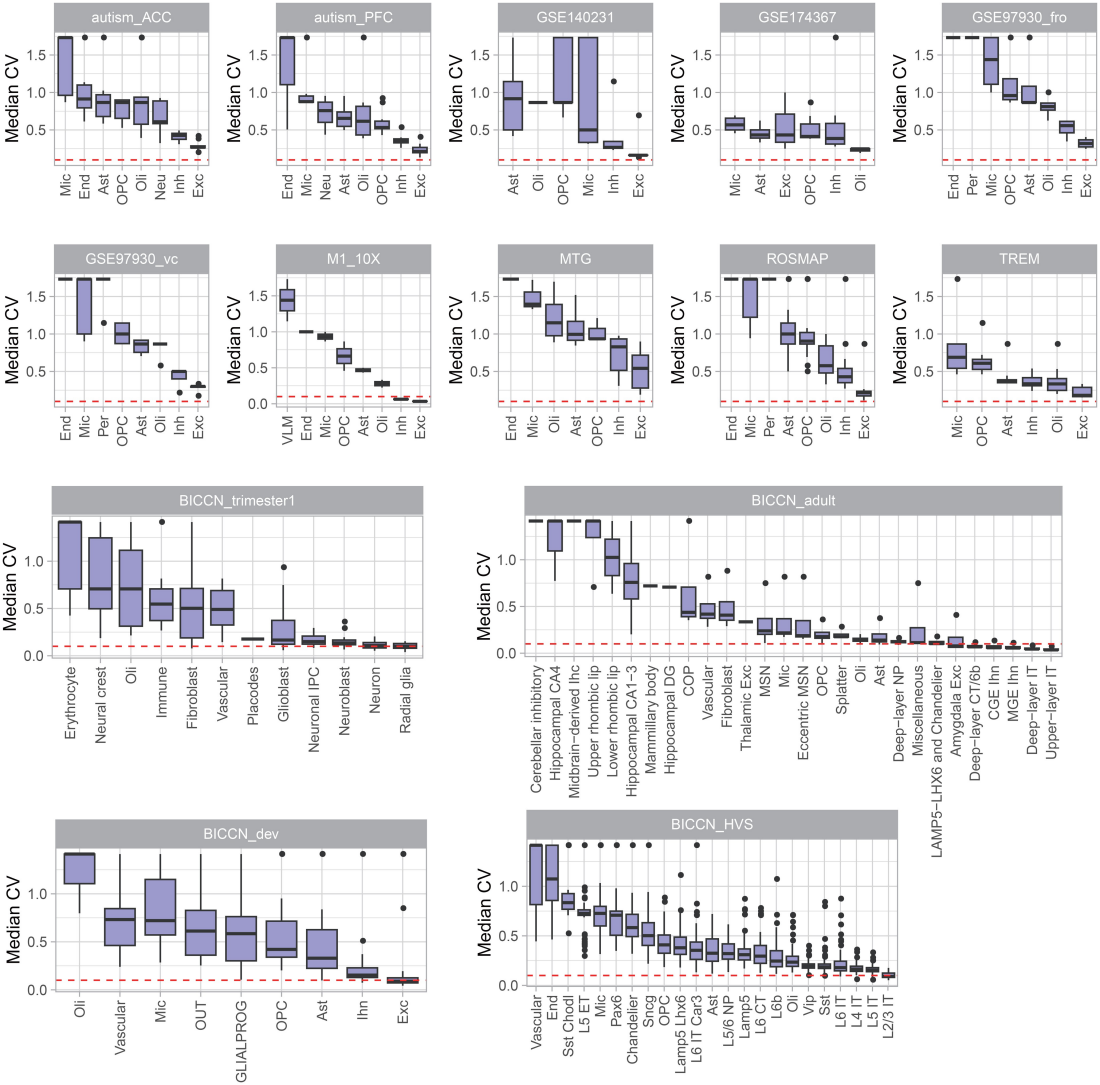
Gene expression precision evaluated by technical replicates in sc/snRNA-seq data Gene expression variability was calculated as the CV of gene expression in three technical replicates based on pseudo-bulks for each gene. The sample with the largest number of total cells in each dataset was used for illustration. The red dashed line denotes the CV threshold of 0.1, which is a threshold recommended for bulk-tissue gene expression quality control processing [16]. CV, coefficient of variation.

Our analysis revealed that the median CV of detected genes across technical replicates based on pseudo-bulks was 0.68 ± 0.24 in the 14 brain datasets (**Figure 3**), much higher than the median CV observed in bulk-tissue RNA-seq [15] (ranging from 0.11 to 0.39) and microarray data [16] (ranging from 0.1 to 0.2). Utilizing cell classification in the BICCN_HVS study, we calculated the CV values at both cell type and subtype levels. The CVs were not significantly different at these two resolution levels, suggesting that the observed variability is not driven by heterogeneity in a higher-level cell classification (Figure S3). A similar pattern was noted in independent mouse brain data when different numbers of cells were sequenced, indicating that low precision is a technical challenge in single-cell data across various sample sources (Figure S4).

To illustrate the expression variability in multiple tissues, the scRNA-seq data from blood, lung, and lymph nodes were evaluated [17]. A similar CV pattern across cell numbers was observed, consistent with findings in brain tissue data. Regardless of tissue type and cell type, approximately 500 cells are needed to drive CV close to 0.1 (Figure S5).

Major cell types exhibited lower CVs than minor cell types. For example, excitatory neurons, as the most abundant cell type, had a median CV of 0.19 ± 0.20 across datasets. In contrast, other cell types had a median CV of 0.55 ± 0.40 across datasets, indicating that the precision problem is particularly severe for low-abundance cell types. Additionally, expression CV was negatively correlated with expression abundance (correlation coefficient = −0.88, *P* < 2.2E−16) (Figure S6A). Notably, marker genes have lower CVs than other genes (Figure S6B).

To compare the expression variability in sc/snRNA-seq data, we evaluated three brain microglia samples with both sc/snRNA-seq data [18]. We observed almost identical CV patterns in the two data types, indicating that quality issues are a common concern for both (Figure S7). We calculated the percentage of samples achieving a designated precision threshold, a CV of 0.1 or lower for each cell type. There was a striking disparity: the proportion of samples from five distinct datasets that satisfied this precision criterion ranged from 3% to 25%, with an average of 5%, as illustrated in Figure S8A. For example, every sample representing upper-layer IT neurons in the Brain Initiative Cell Census Network (BICCN) adult dataset successfully passed the precision assessment (Figure S8B). In the case of the BICCN_HVS dataset, 67% of samples pertaining to IT neurons in layers 2 and 3 met the established quality benchmarks (Figure S8C). Conversely, in the other nine datasets, not a single sample reached the requisite levels of precision. This indicates a prevalent problem with gene expression noise in individual samples of these datasets.

### Expression precision is correlated with the number of cells sequenced

**Figure 4:**
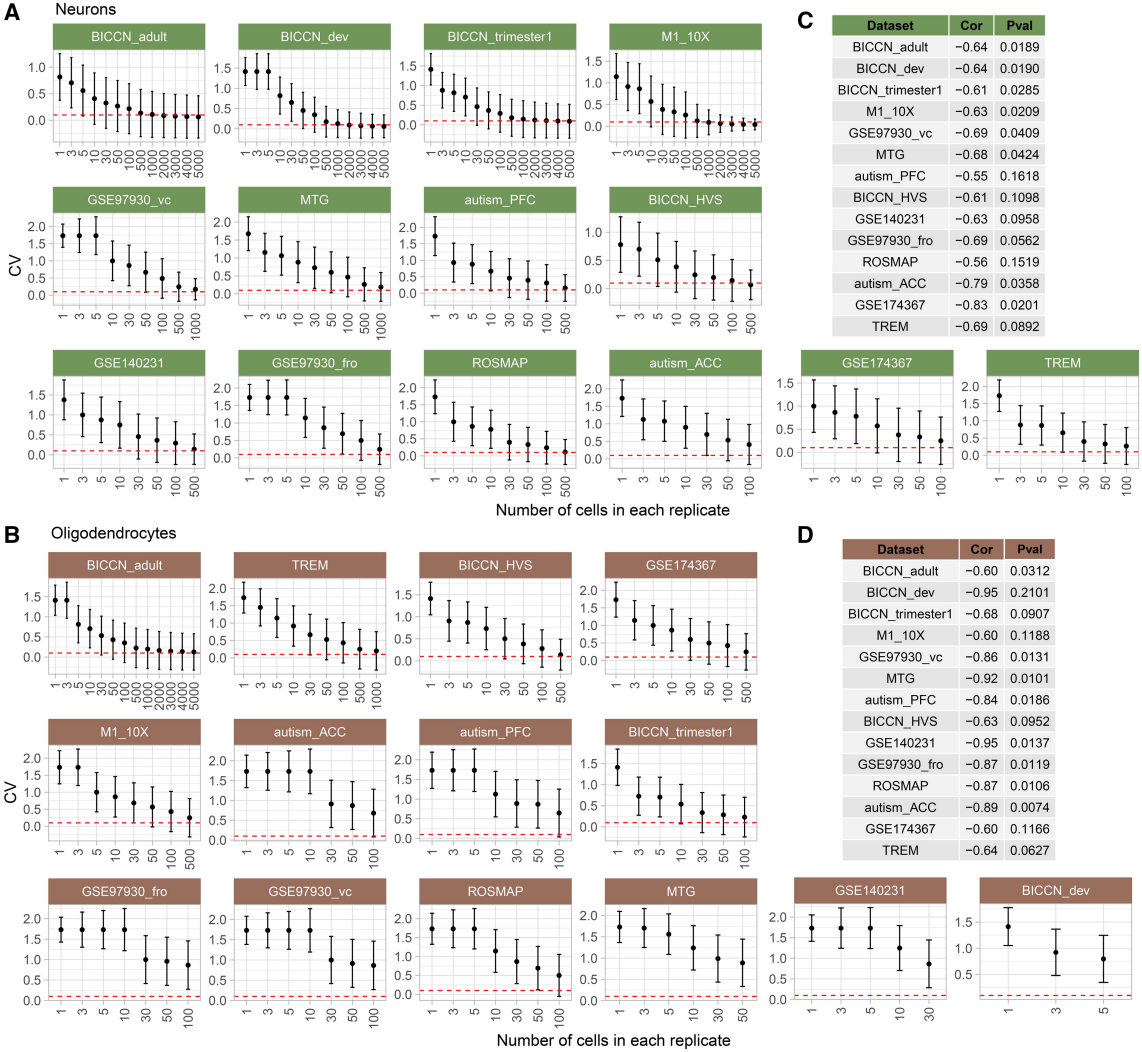
Relationship between cell number and gene expression precision **A.** CV values in downsampled neurons. **B.** CV values in downsampled oligodendrocytes. **C.** Pearson correlation coefficient and *P* value between cell numbers in replicates and median CV in neurons. **D.** Pearson correlation coefficient and *P* value between cell numbers in replicates and median CV in oligodendrocytes. To enhance visual clarity, the number of cells in each replicate was capped at 5000. The Red dashed line denotes a CV threshold of 0.1. Cor, Pearson correlation coefficient; Pval, *P* value.

We expected that the expression precision would be associated with the number of cells sequenced and aimed to identify the minimum cell number for acceptable precision. To prove the expectation by actual data, we generated technical replicates based on pseudo-bulks with varying numbers of cells, ranging from a single cell to the maximum cell number divided by three. The sample with the largest number of total cells in each dataset was utilized for testing. As the number of cells which were pooled into the technical replicates based on pseudo-bulks increased, the overall variability decreased until it reached a plateau for major cell types (**Figure 4**A and B). With the small total number of cells sequenced, the minor cell types did not reach a stable CV (Figure S9). Similar correlation coefficients of −0.66 ± 0.07 and −0.78 ± 0.14 were observed between the number of cells in each replicate and median CV in excitatory neurons and oligodendrocytes, respectively (*P* < 0.05; Figure 4C and D).

The minimum number of cells required for delivering acceptable precision is suggested by data of excitatory neurons. Based on five datasets (BICCN_adult, BICCN_dev, BICCN_trimester1, M1_10X, and BICCN_HVS), approximately 500 cells were required to achieve a median CV close to 0.1 for neurons (Figure 4A). None of other cell types attained CV values as low as 0.1 and they all had fewer than 500 cells sequenced.

### RNA integrity is correlated with expression precision

The cell numbers required for achieving an acceptable precision level in sc/snRNA-seq data vary across studies, suggesting that expression precision may not be solely dependent on the number of cells sequenced. We examined the effects of four technical factors, including RNA integrity, sequencing depth, sequencing saturation, and sequencing platform, on expression precision of excitatory neurons.

**Figure 5:**
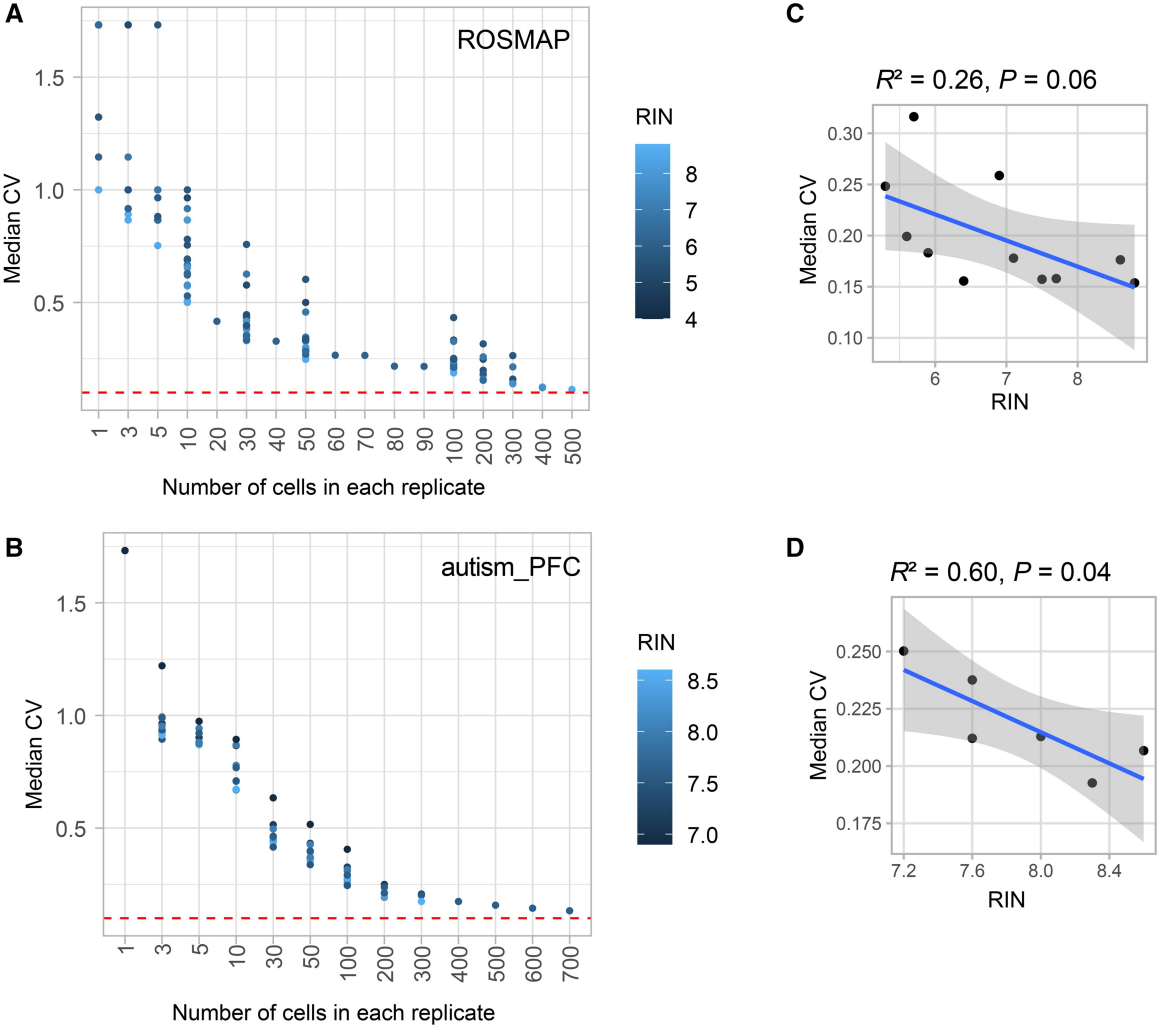
Association between RNA integrity and gene expression variability across technical replicates in snRNA-seq data **A.** and **B.** Relationship between number of cells in replicates and median CV was tested in ROSMAP (A) and autism_PFC (B) datasets. Samples were colored by RIN. Red dashed line denotes the CV threshold of 0.1. **C.** and **D.** Relationship between RIN and median CV when replicate contains 200 cells in ROSMAP (C) and autism_PFC (D) datasets. Linear regression model was used. RIN, RNA integrity number; ROSMAP, Religious Order Study and Memory and Aging Project.

We tested the relationship between RNA integrity, as measured by the RNA integrity number (RIN), and median CV in technical replicates. Two datasets with RIN information available for analysis were used. Samples with higher RIN tended to have lower CV (**Figure 5**A and B). By zooming into replicates with 200 cells, negative correlations were observed between RIN and median CV in the ROSMAP dataset (*R*^2^ = 0.26, *P* = 0.06; Figure 5C) and the autism_PFC dataset (*R*^2^ = 0.60, *P* = 0.04; Figure 5D), suggesting that RNA integrity is another factor contributing to expression precision.

In the autism_PFC dataset, we also explored the correlation between median CV and total sequencing depth (*P* = 0.89) and saturation rates (*P* = 0.76), but no significant correlation was found (Figure S10A and B). We also compared expression variability across technical replicates in data generated from two different sequencing platforms, 10X Chromium (autism_PFC) and Smart-seq (MTG). The median CV across detected genes in replicates constructed by 200 cells was used for comparison. No significant difference in gene expression variability was observed between the two technologies (*P* = 0.56, Wilcoxon signed-rank test; Figure S10C), indicating that the precision problem is not unique to a specific sequencing platform.

### Low expression accuracy in scRNA-seq data is associated with the number of cells sequenced

**Figure 6:**
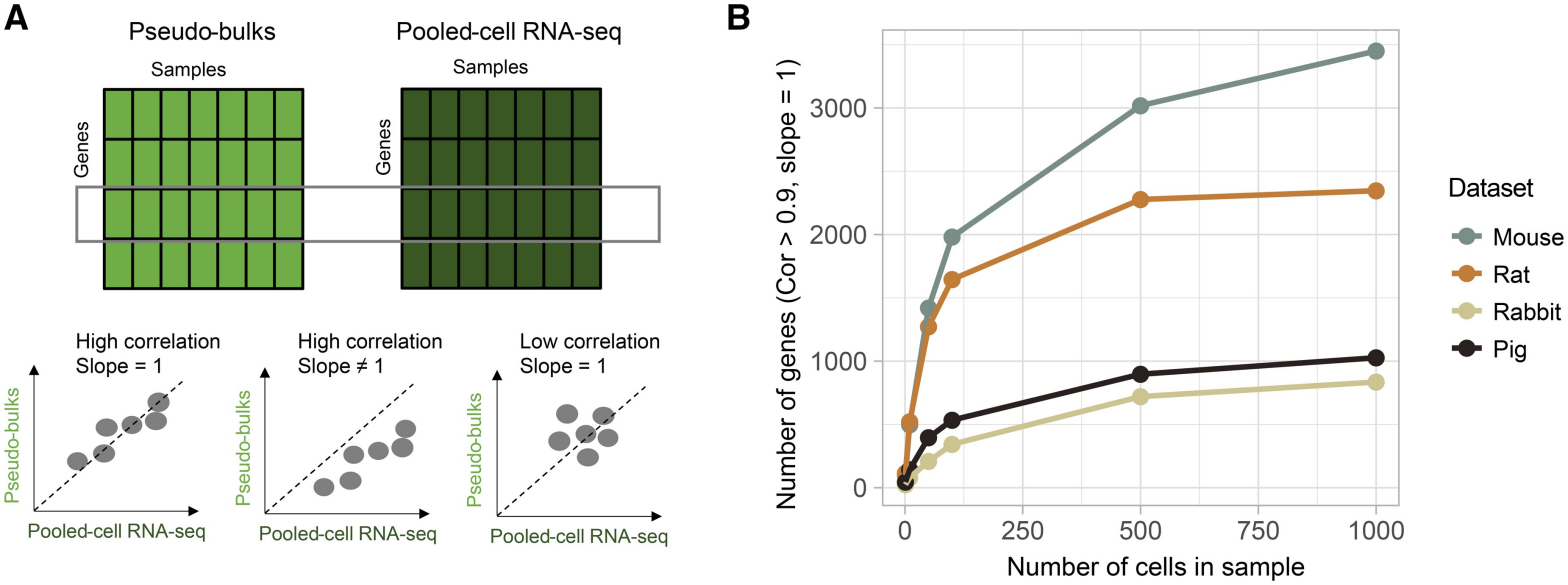
Relationship between expression accuracy and cell number **A.** Illustration of expression accuracy assessment. **B.** Relationship between cell number and number of genes with good accuracy defined by Pearson correlation and linear regression model.

To evaluate the accuracy of gene expression, we compared pooled-cell RNA-seq data with scRNA-seq data of cultured mononuclear phagocytes from matched samples (**Figure 6**A). RNA-seq data from pooled cultured cells (of one type) were referred to as pooled-cell RNA-seq. The gene expression levels from pooled-cell RNA-seq were considered as the ground truth. We used Pearson correlation and linear regression to assess the expression accuracy. In the linear regression model, the ground truth was treated as the independent variable, while the pseudo-bulks from sample-matched scRNA-seq were the dependent variable. We tested the significance of the slope deviating from one. The significance of the linear regression, combined with the Pearson correlation coefficient, was used to measure expression accuracy. We calculated the expression accuracy independently for each of the four species. To illustrate the relationship between the number of cells sequenced and expression accuracy, we performed downsampling experiments, ranging from 1000 to 1 cell for each sample.

The number of genes with good accuracy decreased in downsampling (Figure 6B). We observed 3450 out of 13,907 detected genes with good accuracy as defined by the criteria of regression slope of 1 (*P* = 0.05) and correlation coefficient of 0.9 when 1000 cells were analyzed for each sample in mouse data. When each sample contained a single cell, only 100 genes showed good accuracy. When the data have 500 cells in each sample, the gene accuracy tends to reach a stable value. Similar patterns were observed in data from rat, pig, and rabbit, though pig and rabbit data showed overall worse performance than mouse and rat data (Table S1).

The relationship between the number of cells sequenced and expression accuracy was replicated in the simulation data. In the simulation, scRNA-seq data of six samples, each with 3000 cells, were synthesized. The pseudo-bulks of 3000 cells in each sample were used as ground truth. We observed that the number of genes with good accuracy increased with larger cell numbers (Figure S11), consistent with results from our real data. Notably, when at least 500 cells were sampled, the number of genes with good accuracy began to stabilize.

### Noise level and trait effect size interactively affect the reproducibility of DE analysis in scRNA-seq data

**Figure 7:**
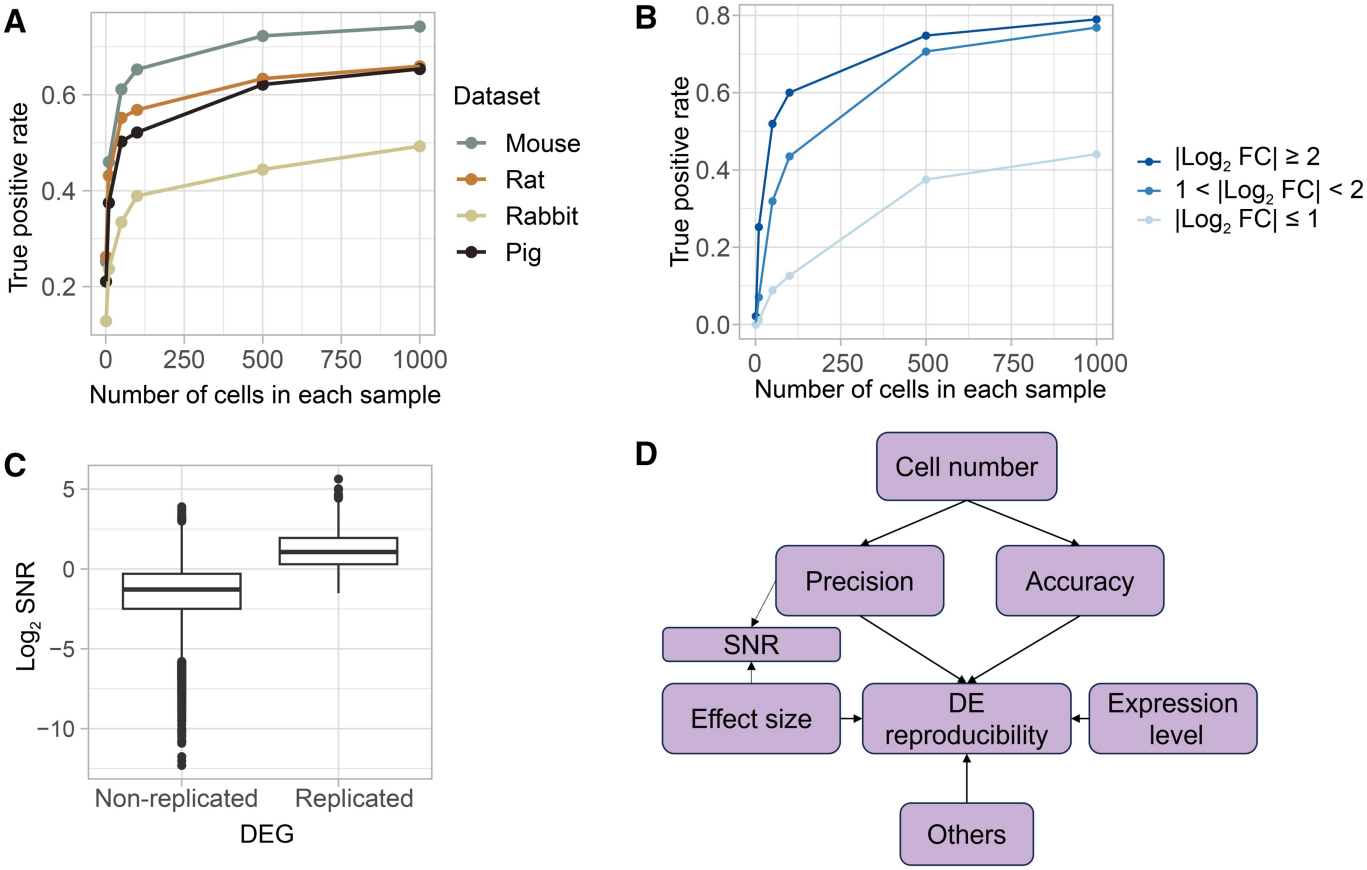
Reproducibility of DE analysis in scRNA-seq data **A.** True positive rate of DEGs in data with different numbers of cells. Datasets from different species are colored. **B.** True positive rate of DEGs with different effect sizes categorized by log_2_ FC of DE. **C.** Relationship between SNR and DEG reproducibility in mouse data. SNR is defined as normalized effect size divided by CV. **D.** Model of DE reproducibility. SNR, signal-to-noise ratio; DEG, differentially expressed gene.

To assess the impact of data quality on downstream analysis, we conducted a DE analysis in sample-matched scRNA-seq and pooled-cell RNA-seq datasets independently, comparing lipopolysaccharide (LPS)-treated and untreated groups using the edgeR algorithm. Genes were considered significantly differentially expressed genes (DEGs) when their false discovery rate (FDR)-corrected *P* value was less than 0.05. Since we already showed that both expression precision and accuracy were positively correlated with the number of cells sequenced, we employed a downsampling strategy to investigate the influence of cell number on DE results. By utilizing the DE results in pooled-cell RNA-seq data as ground truth, we evaluated the overall reproducibility of DE results in scRNA-seq data by calculating the true positive rate — the proportion of actual positives that are correctly identified as positive. Notably, as the number of cells increased, the true positive rate improved and had a plateau at about 500 cells (**Figure 7**A). The true positive rates were 0.72, 0.63, 0.62, and 0.44 in data from mouse, rat, pig, and rabbit, when 500 cells were included in each sample.

Effect size, which reflects the differences between the compared groups, plays a crucial role in determining the statistical power of a DE analysis. By categorizing the mouse DEGs into three groups based on effect size: high [|log_2_ fold change (FC)| ≥ 2], medium (1 < |log_2_ FC| < 2), and low (|log_2_ FC| ≤ 1), we observed that DEGs with high and medium effect sizes demonstrated a better true positive rate than those with low effect sizes. DEGs with medium effect sizes still exhibited a relatively lower true positive rate compared to genes with high effect sizes, particularly when the number of cells was limited (Figure 7B). For example, when 500 cells were included in each sample, DEGs with high effect sizes (over two-fold changes) had a true positive rate of 0.73, whereas DEGs with low effect sizes had a true positive rate of only 0.38. When only 50 cells were included in each sample, the true positive rates were 0.41 and 0.09 for DEGs with high and low effect sizes, respectively.

This suggests that the relationship between effect size and noise level has an interactive impact on DEG reproducibility. To quantify this combined effect, we adopted signal-to-noise ratio (SNR) metric for each gene, defined as normalized effect size divided by CV. Using mouse data as an example, we found that replicated DEGs exhibited significantly higher SNRs than non-replicated DEGs (*P* < 2.2 × 10^−16^) (Figure 7C). This trend was consistent with the observation that DEGs showing higher expression tend to have better reproducibility (Figure S12). The factors influencing DEG reproducibility are summarized in Figure 7D.

To evaluate the applicability of the 500-cell cutoff and the SNR measurement, we applied various cell number cutoffs to an independent dataset from Ruzicka and colleagues [9], which conducted DE analysis on two schizophrenia postmortem brain cohorts (MCL and Mt Sinai). Using an exact test, we assessed the reproducibility of DEGs across the two cohorts. At a 500-cell cutoff, cell types with significant DEG reproducibility were clearly distinguishable from those without (Figure S13A). However, reducing the cell number cutoff to lower thresholds, such as 300 or 100 cells — commonly regarded as acceptable in practice — may result in misleading indications of reproducibility. For instance, the Vip neuron emerged as a potential cell type with replicable DEGs at these lower cutoffs, yet its reproducibility was not statistically significant. Moreover, we found that reproduced DEGs exhibited significantly higher SNR (*P* = 0.0002) (Figure S13B), effectively distinguishing reproducible genes from non-reproducible ones in this dataset.

## Discussion

The use of sc/snRNA-seq in biological studies has become a common practice, necessitating meticulous evaluation of data quality to avoid misleading or even false findings. The current investigation assesses the expression precision and accuracy of published sc/snRNA-seq data. By analyzing 23 representative datasets, we demonstrated that the gene expression per individual measured for most cell types was of low precision and accuracy. We found a robust correlation between the number of cells sequenced and the precision, accuracy, and reproducibility of downstream DE analysis. Only cell types having a large number of cells (minimum 500 cells) sequenced delivered relatively accurate and precise quantification of gene expression and, consequently, credible results of downstream analyses, such as case–control comparisons.

Many studies have speculated that cell number, RNA integrity, and sequencing depth influence sc/snRNA-seq data quality, but none have systematically quantified these effects across datasets or established actionable thresholds. This lack of clear, reproducible standards has led to inconsistencies in experimental design and, in some cases, unreliable — or even outright incorrect — conclusions. A striking example comes from Murphy et al. [19], who demonstrated that many of the transcriptional differences reported by Mathys et al. [8] regarding Alzheimer’s disease were false positives attributable to inadequate noise control and flawed DE analysis. Alarmingly, despite its misidentified genes, the study by Mathys et al. has been cited over 2000 times [20–22], significantly shaping the Alzheimer’s research landscape. This is just one example; similar issues permeate the field [23–25]. This concern aligns with the findings of previous studies [26,27]. It is urgent that the single-cell research community recognizes the critical importance of data quality to prevent misleading findings and ensure the reliability of future discoveries. Our study addresses this urgent need by providing a quantitative threshold driven by large datasets, gene-level evaluation metrics, and practical tools and guidance.

Our study establishes quantitative thresholds critical for ensuring high-quality single-cell data and results. Prior studies qualitatively recognized that increasing the number of cells improves data quality and reproducibility [11,28], but the relationship between them is non-linear and the gene expression precision, accuracy, and reproducibility saturate at certain cell numbers (Figures 4A, 6B, and 7A). Therefore, a quantitative cutoff is required to exclude low-quality genes and samples, similar to standard practices in bulk RNA-seq. This cutoff has never been defined, creating a gap that limits consistency and reliability in single-cell studies. Our systematic evaluation of 23 sc/snRNA-seq datasets of mature cell types from brain and other tissues demonstrates that at least 500 cells per cell type per individual are required for robust measurements — an evidence-based threshold previously missing in the field.

The criteria used for evaluating expression precision in this study are standard statistical techniques. We used CV < 0.1 as the cutoff for the expression precision in this evaluation. This is based on previous quality evaluations of bulk RNA-seq data [16]. We believe that holding sc/snRNA-seq data to the same standard as bulk RNA-seq is appropriate and lowering the standard will lead to noisy results and poor reproducibility. Additionally, our evaluation demonstrates that CV tends to stabilize at around 0.1 in single-cell data, as the number of cells increases. However, when constructing the technical replicates based on pseudo-bulks, we assumed homogeneity within one cell type. Such an assumption can be violated by heterogeneity caused by cell subtypes and states, which may explain the minimum CV that is observed. Nonetheless, our results indicate that cell subtype is not the major cause of poor precision in the cell types that we evaluated, as the precision at the cell-type level is not worse than that at subtype level.

RNA quality is another crucial factor impacting expression precision. Notably, in the BICCN_HVS study [29], the minimum number of cells required to achieve a CV of 0.1 was 500, but other datasets require even larger numbers of cells. A key factor contributing to this difference may be the use of surgical samples in the BICCN_HVS study, as these samples tend to be less degraded than frozen postmortem brain samples. Ensuring high RNA quality in samples, such as using RNA with RIN values greater than 7, will likely reduce the number of cells required for quality quantification.

The SNR emerges as a pivotal determinant of the reproducibility of DE analysis. Our investigation revealed that DEGs with large effect sizes exhibit superior reproducibility compared to those with smaller effect sizes. When 500 cells were included in each sample where the noise level was low, DEGs with large effect sizes had a true positive rate of 0.73, whereas DEGs with small effect sizes had a true positive rate of only 0.38. When the cell number decreased to 50 cells where the noise level was high, the true positive rates were 0.41 and 0.09 for DEGs with large and small effect sizes, respectively. This comparison indicates that the technical noise matters more for the smaller biological effects and technical noise may be manageable for phenotypes associated with pronounced expression changes. Improving data quality becomes more critical in scenarios where the effect size of the phenotype approaches the noise level. This is particularly relevant for many complex diseases, including neuropsychiatric disorders, where the effect size is typically small [30], necessitating an increase in the number of cells to minimize technical noise.

This work provides gene-level metrics to help refine reliable signals. Most prior studies assessed data quality at the cell or sample level, which can be biased by highly expressed genes. In contrast, our study introduces a gene-by-gene evaluation framework, enabling precise quality and reproducibility assessments for individual genes — a crucial advancement for downstream analyses like DE. Specifically, we introduce the SNR as a key metric for assessing DEG reproducibility, calculated by dividing fold change by CV. Applying this approach to schizophrenia DEGs and mouse data, we found that reproduced DEGs have significantly higher SNR, which effectively distinguishes reproducible genes from non-reproducible ones in this data, providing a practical metric for ensuring reliable single-cell data analysis.

We introduce VICE, a powerful tool that enables researchers to assess the quality of existing single-cell data and predict the reliability of DE results. With calculated CV values, users can: (1) determine noise levels across different cell types and samples; and (2) identify genes with low noise levels, ensuring that only high-confidence genes are prioritized for analysis. By inputting cell numbers, effect sizes, and noise levels, VICE estimates the true positive rate for single-cell DE analysis, providing a direct, data-driven framework for optimizing experimental design and result interpretation. For trait-specific analyses, such as DE, VICE can be used to: (1) estimate the true positive rate based on sample size and cell number to guide study design; and (2) evaluate DEG reliability by estimating the true positive rate based on signal-to-noise levels rather than relying solely on *P* values.

We provide the following guidelines for future single-cell research. For general data quality control, we recommend: (1) prioritizing high-quality RNA samples (RIN ≥ 7) whenever possible, as degraded RNA increases noise and reduces reproducibility; and (2) ensuring sufficient cell numbers for reliable analysis. We suggest at least 500 cells per cell type per individual for optimal precision. If this is not feasible, focusing on genes with low noise levels is advisable. Adjusting the CV threshold based on trait effect size can help balance precision with dataset constraints. For result reporting, we recommend: (1) routinely reporting CV values and associated power as quality metrics in single-cell data analysis; and (2) providing the median CV for each sample in sc/snRNA-seq experiments to assess sample quality. These benchmarks set optimal standards rather than rigid requirements. Researchers can adapt them as needed, using pseudo-bulk strategies for low cell numbers or adjusting CV thresholds based on effect size. Our approach provides practical, data-driven guidance that supports informed decision-making rather than imposing one-size-fits-all rules.

Our findings hold significant implications across multiple domains. Many researchers have reported results from minor cell types in a variety of tissues, but our work casts doubt about the validity of conclusions drawn from much of these studies due to insufficient numbers of cells. The accurate identification and comprehensive study of these minor cell types necessitates sequencing more cells. Beyond elucidating the nuances of DE analysis, our results imply that low precision and accuracy impact other analytical methodologies, including cell classification [31,32], expression quantitative trait locus (eQTL) mapping [33], and the construction of co-expression networks [34]. The effect of cell numbers on other data analyses remains to be explored.

Our study rigorously quantifies these effects and provides concrete, data-driven guidelines to improve sc/snRNA-seq studies. Our goal is not to rescue poor experimental designs — there is no simple fix for flawed data. Instead, we define the scale of the problem with precise numbers, highlighting critical pitfalls in single-cell data analysis. We equip researchers with clear, quantitative metrics to assess which genes and samples meet quality standards for reliable downstream analysis. More importantly, we provide practical tools and data-driven cutoffs, ensuring that future studies are designed correctly from the start, minimizing errors, and maximizing reproducibility.

## Conclusion

In this study, we conducted a quantitative evaluation of expression precision and accuracy across 23 representative sc/snRNA-seq datasets, revealing significant deficiencies in gene expression measurements — particularly when sequencing a limited number of cells. We demonstrate that the reproducibility of DE analysis is tightly correlated with cell number, emphasizing the need for data-driven thresholds in study design. To improve the reliability and reproducibility of sc/snRNA-seq studies, we recommend sequencing at least 500 cells per cell type per individual, including minor cell types and RNA quality (RIN ≥ 7). Recognizing practical constraints, we provide flexible, evidence-based guidelines rather than rigid requirements. We strongly advocate for quality assessment before downstream analyses to prevent false discoveries. To facilitate this, we developed VICE, a tool that quantifies technical variability, estimates the true positive rate of DE results, and enables data-driven decision-making in sc/snRNA-seq studies.

## Materials and methods

### Collection of sc/snRNA-seq data from cortex

A total of 14 brain sc/snRNA-seq datasets were obtained for analysis. The collected datasets were derived from human brain studies published between 2012 and 2023 [7,8,35–42]. Samples from individuals with brain disorders were excluded from the analysis to prevent biasing the expression profiles. The raw count and cell annotation data were obtained from the original studies. Due to differences in cell classification across studies, we harmonized cell identities into eight major cell types present in the adult brain, namely excitatory neurons, inhibitory neurons, oligodendrocytes, oligodendrocyte precursor cells, astrocytes, microglia, endothelial cells, and pericytes. The annotation of data from BICCN 2023 collection was retained to evaluate cell subtypes. The scRNA-seq data of blood, lung, and lymph node from Tabula Sapiens consortium were used for evaluating expression variability in multiple tissues [17].

### Collection of sample-matched data from four species

To assess expression accuracy, we utilized four sample-matched scRNA-seq and pooled-cell RNA-seq datasets. These datasets were sourced from Hagai and colleagues [43], encompassing bone marrow-derived mononuclear phagocytes derived from mouse, rat, pig, and rabbit, all subjected to stimulation with either lipopolysaccharide or poly-I:C for a duration of 4 h. Within each species, a total of three samples received lipopolysaccharide treatment, while three additional samples were designated as control groups. We employed the preprocessed data provided by Squair and colleagues [26], which are available at https://doi.org/10.5281/zenodo.5048449.

### RIN

The RIN is a numerical value that measures the quality of RNA samples. It is calculated before sequencing using an automated analysis of RNA molecules through electrophoresis, such as Agilent 2100 bioanalyzer. The RIN ranges from 1 to 10, with 10 indicating fully intact RNA and 1 indicating completely degraded RNA. We obtained the RIN of samples from the original studies.

### Processing of sc/snRNA-seq data

The sc/snRNA-seq data underwent processing using Seurat (v4) [44]. The raw count matrix and cell annotation matrix were used as input to Seurat. We filtered out genes with zero expression in more than 1/1000 of the total cells in each dataset. The proportion of transcripts mapped to mitochondrial genes was calculated for each cell, and cells with 10% or more mitochondrial gene expression were removed to prevent the inclusion of dead cells. Additionally, cells with less than 200 detected genes or those with more than three standard deviations from the mean number of detected genes were excluded. The count data were normalized based on library size and were scaled with a factor of 10,000. The normalized data were then log-transformed.

### Marker gene identification

To identify marker genes in the Religious Order Study and Memory and Aging Project (ROSMAP) dataset, we utilized a one *versus* second high strategy at both the cell and pseudo-bulk level. At the cell level, marker genes were identified using Seurat. Genes with a proportion of zero expression greater than 15% in the target cell type were removed prior to marker gene identification. The Wilcoxon signed-rank test was used to assess the expression difference, and genes with a log_2_ FC greater than 1 and FDR-corrected *P* value less than 0.05 were defined as marker genes. At the pseudo-bulk level, pseudo-bulks were constructed by aggregating gene expression for the same cell type from the same individual. Marker genes were then tested using DESeq2 [45], and the likelihood ratio test was utilized to evaluate the expression difference between the two cell groups. Marker genes with a log_2_ FC greater than 2 and FDR-corrected *P* value less than 0.05 were defined as marker genes at the pseudo-bulk level. Finally, marker genes supported by both cell-level and pseudo-bulk-level tests were selected as final marker genes.

### Technical replicate construction and CV calculation

To generate technical replicates based on pseudo-bulks for each cell type, cells in the count matrix were randomly divided into three groups for the same individual. The count expression for each gene was then summed within each group. The CV value was calculated for each gene as follows:


(1) $${\mathrm{CV}}_{i}=\frac{sd(x)}{\mathrm{mean}(x)}$$

where *x* represents the gene expression of gene *i* across three replicates of a specific cell type. To ensure the robustness of technical replicates, the cell groupings and CV calculations were repeated 100 times, and the average CV across 100 samplings was used.

### CV–cell number relationship in data with cell class and subclass annotations

To compare the relationship between CV values and the number of sequenced cells in the mouse class and subclass data, a Student’s *t*-test was used (Figure S3). To compare the relationship between CV values and the number of sequenced cells in the mouse class and subclass data when different number of cells were sequenced, we used a two-sample Z-test (Figure S4). The null hypothesis was that the slope in the regression model testing the relationship between the number of cells and CV values was the same in the class and subclass data. We calculated the difference in slope between the two datasets as follows:


(2) $$\mathrm{diff}=\frac{b1-b2}{\sqrt{{se1}^{2}+{se2}^{2}}}$$

where *b*1 and *b*2 were the coefficients, and *se*1 and *se*2 were the standard errors from the regression model in the class and subclass data, respectively. We then used the area of the standard normal curve corresponding to the calculated difference to determine the probability in a two-tailed manner.

### Data simulation

Single-cell count data were simulated based on a negative binomial model using the R package Splatter [46]. Two conditions were generated with the “group” simulation, with between 10 and 3000 cells per sample and three replicates per condition. The proportion of differentially expressed genes (de.prob) was set to 0.25.

### Evaluation of expression accuracy

Since no single statistic is sufficient to describe accuracy, we developed a composite criterion that captures the bias and distance from ground truth simultaneously using Pearson correlation and a linear regression model. The scRNA-seq data were summarized by pseudo-bulks first. The pseudo-bulks were normalized by the library size and were transformed into log_2_-transformed counts per million (CPM). Then the batch effect between pseudo-bulks and pooled-cell RNA-seq data was corrected using the combat function in the sva package [47]. Pearson correlation between sample-matched scRNA-seq and pooled-cell RNA-seq was calculated for each gene. In the linear regression model, the expression in pooled-cell RNA-seq was treated as the independent variable, and the expression in scRNA-seq was treated as the dependent variable. The intercept of linear regression model was set to 0. By setting offset in function lm in R, the significance of slope deviating from 1 was tested. Good accuracy was defined as correlation coefficient over 0.9 and *P* value of linear regression over 0.05.

### DE analysis

DE analysis was carried out on both scRNA-seq and pooled-cell RNA-seq datasets to examine the expression disparities between samples treated with lipopolysaccharide and the control samples. For scRNA-seq data, DE analysis was performed on pseudo-bulk data using the likelihood ratio test approach provided by edgeR [48]. For pooled-cell RNA-seq data, edgeR was performed on the count data directly. Genes exhibiting an FDR-corrected *P* value of less than 0.05 were classified as DEGs. We assessed the consistency between DE results obtained from single-cell and pooled-cell RNA-seq using true positive rate (*i.e.*, the proportion of DEGs identified in pooled-cell RNA-seq that were also replicated in the scRNA-seq data).

### Application of DE analysis to schizophrenia postmortem brain cohorts

The schizophrenia DE results were obtained from the supplementary materials of Ruzicka and his colleagues [9]. This study includes samples from the Mt Sinai and MCL cohorts. DEGs were defined as genes with log_2_ FC > 0.1 and FDR < 0.05. Replicated DEGs were those that met the DE criteria in both cohorts. The exact test [49] was performed to assess the statistical significance of DEG replication.

## Code availability

The code for this study can be accessible at https://github.com/RujiaDai/VICE. The code has also been submitted to BioCode at the National Genomics Data Center (NGDC), China National Center for Bioinformation (CNCB) (BioCode: BT007673), which is publicly accessible at https://ngdc.cncb.ac.cn/biocode/tool/BT7673.

## CRediT author statement

**Rujia Dai:** Conceptualization, Formal analysis, Data curation, Validation, Software, Methodology, Writing – original draft. **Ming Zhang:** Formal analysis, Data curation, Validation, Software, Methodology. **Tianyao Chu:** Formal analysis, Data curation, Validation, Software, Methodology. **Richard Kopp:** Investigation, Writing – review & editing. **Chunling Zhang:** Investigation, Writing – review & editing. **Kefu Liu:** Investigation, Writing – review & editing. **Yue Wang:** Investigation, Writing – review & editing. **Xusheng Wang:** Investigation, Writing – review & editing. **Chao Chen:** Funding acquisition, Supervision, Project administration, Conceptualization. **Chunyu Liu:** Funding acquisition, Supervision, Project administration, Conceptualization, Writing – original draft. All authors have read and approved the final manuscript.

## Competing interests

The authors have declared no competing interests.

## Supplementary Material

qzaf077_Supplementary_Data

## References

[1] Tang F , Barbacioru C, Wang Y, Nordman E, Lee C, Xu N, et al mRNA-Seq whole-transcriptome analysis of a single cell. Nat Methods 2009;6:377–82. 19349980 10.1038/nmeth.1315

[2] Svensson V , Vento-Tormo R, Teichmann SA. Exponential scaling of single-cell RNA-seq in the past decade. Nat Protoc 2018;13:599–604. 29494575 10.1038/nprot.2017.149

[3] Cuomo ASE , Nathan A, Raychaudhuri S, MacArthur DG, Powell JE. Single-cell genomics meets human genetics. Nat Rev Genet 2023;24:535–49. 37085594 10.1038/s41576-023-00599-5 PMC10784789

[4] BRAIN Initiative Cell Census Network. A multimodal cell census and atlas of the mammalian primary motor cortex. Nature 2021;598:86–102. 34616075 10.1038/s41586-021-03950-0 PMC8494634

[5] Eze UC , Bhaduri A, Haeussler M, Nowakowski TJ, Kriegstein AR. Single-cell atlas of early human brain development highlights heterogeneity of human neuroepithelial cells and early radial glia. Nat Neurosci 2021;24:584–94. 33723434 10.1038/s41593-020-00794-1 PMC8012207

[6] Nowakowski TJ , Bhaduri A, Pollen AA, Alvarado B, Mostajo-Radji MA, Di Lullo E, et al Spatiotemporal gene expression trajectories reveal developmental hierarchies of the human cortex. Science 2017;358:1318–23. 29217575 10.1126/science.aap8809 PMC5991609

[7] Velmeshev D , Schirmer L, Jung D, Haeussler M, Perez Y, Mayer S, et al Single-cell genomics identifies cell type-specific molecular changes in autism. Science 2019;364:685–9. 31097668 10.1126/science.aav8130 PMC7678724

[8] Mathys H , Davila-Velderrain J, Peng Z, Gao F, Mohammadi S, Young JZ, et al Single-cell transcriptomic analysis of Alzheimer’s disease. Nature 2019;570:332–7. 31042697 10.1038/s41586-019-1195-2 PMC6865822

[9] Ruzicka WB , Mohammadi S, Fullard JF, Davila-Velderrain J, Subburaju S, Tso DR, et al Single-cell multi-cohort dissection of the schizophrenia transcriptome. Science 2024;384:eadg5136. 38781388 10.1126/science.adg5136 PMC12772489

[10] Bacher R , Kendziorski C. Design and computational analysis of single-cell RNA-sequencing experiments. Genome Biol 2016;17:63. 27052890 10.1186/s13059-016-0927-y PMC4823857

[11] Lahnemann D , Koster J, Szczurek E, McCarthy DJ, Hicks SC, Robinson MD, et al Eleven grand challenges in single-cell data science. Genome Biol 2020;21:31. 32033589 10.1186/s13059-020-1926-6 PMC7007675

[12] Brennecke P , Anders S, Kim JK, Kolodziejczyk AA, Zhang X, Proserpio V, et al Accounting for technical noise in single-cell RNA-seq experiments. Nat Methods 2013;10:1093–5. 24056876 10.1038/nmeth.2645

[13] Hicks SC , Townes FW, Teng M, Irizarry RA. Missing data and technical variability in single-cell RNA-sequencing experiments. Biostatistics 2018;19:562–78. 29121214 10.1093/biostatistics/kxx053 PMC6215955

[14] Wu AR , Neff NF, Kalisky T, Dalerba P, Treutlein B, Rothenberg ME, et al Quantitative assessment of single-cell RNA-sequencing methods. Nat Methods 2014;11:41–6. 24141493 10.1038/nmeth.2694 PMC4022966

[15] Li S , Tighe SW, Nicolet CM, Grove D, Levy S, Farmerie W, et al Multi-platform assessment of transcriptome profiling using RNA-seq in the ABRF next-generation sequencing study. Nat Biotechnol 2014;32:915–25. 25150835 10.1038/nbt.2972 PMC4167418

[16] Consortium M , Shi L, Reid LH, Jones WD, Shippy R, Warrington JA, et al The MicroArray Quality Control (MAQC) project shows inter- and intraplatform reproducibility of gene expression measurements. Nat Biotechnol 2006;24:1151–61. 16964229 10.1038/nbt1239 PMC3272078

[17] Tabula Sapiens C , Jones RC, Karkanias J, Krasnow MA, Pisco AO, Quake SR, et al The Tabula Sapiens: a multiple-organ, single-cell transcriptomic atlas of humans. Science 2022;376:eabl4896. 35549404 10.1126/science.abl4896 PMC9812260

[18] Thrupp N , Sala Frigerio C, Wolfs L, Skene NG, Fattorelli N, Poovathingal S, et al Single-nucleus RNA-seq is not suitable for detection of microglial activation genes in humans. Cell Rep 2020;32:108189. 32997994 10.1016/j.celrep.2020.108189 PMC7527779

[19] Murphy AE , Fancy N, Skene N. Avoiding false discoveries in single-cell RNA-seq by revisiting the first Alzheimer’s disease dataset. Elife 2023;12:RP90214. 10.7554/eLife.90214 PMC10695556 38047913

[20] Agarwal D , Sandor C, Volpato V, Caffrey TM, Monzon-Sandoval J, Bowden R, et al A single-cell atlas of the human substantia nigra reveals cell-specific pathways associated with neurological disorders. Nat Commun 2020;11:4183. 32826893 10.1038/s41467-020-17876-0 PMC7442652

[21] Zhou Y , Song WM, Andhey PS, Swain A, Levy T, Miller KR, et al Human and mouse single-nucleus transcriptomics reveal TREM2-dependent and TREM2-independent cellular responses in Alzheimer’s disease. Nat Med 2020;26:131–42. 31932797 10.1038/s41591-019-0695-9 PMC6980793

[22] Morabito S , Miyoshi E, Michael N, Shahin S, Martini AC, Head E, et al Single-nucleus chromatin accessibility and transcriptomic characterization of Alzheimer’s disease. Nat Genet 2021;53:1143–55. 34239132 10.1038/s41588-021-00894-z PMC8766217

[23] Lau SF , Cao H, Fu AKY, Ip NY. Single-nucleus transcriptome analysis reveals dysregulation of angiogenic endothelial cells and neuroprotective glia in Alzheimer’s disease. Proc Natl Acad Sci U S A 2020;117:25800–9. 32989152 10.1073/pnas.2008762117 PMC7568283

[24] Soreq L , Bird H, Mohamed W, Hardy J. Single-cell RNA sequencing analysis of human Alzheimer’s disease brain samples reveals neuronal and glial specific cells differential expression. PLoS One 2023;18:e0277630. 36827281 10.1371/journal.pone.0277630 PMC9955959

[25] Liu CS , Park C, Ngo T, Saikumar J, Palmer CR, Shahnaee A, et al RNA isoform diversity in human neurodegenerative diseases. eNeuro 2024;11:ENEURO.0296-24.2024. 10.1523/ENEURO.0296-24.2024 PMC11693435 39658200

[26] Squair JW , Gautier M, Kathe C, Anderson MA, James ND, Hutson TH, et al Confronting false discoveries in single-cell differential expression. Nat Commun 2021;12:5692. 34584091 10.1038/s41467-021-25960-2 PMC8479118

[27] Soneson C , Robinson MD. Bias, robustness and scalability in single-cell differential expression analysis. Nat Methods 2018;15:255–61. 29481549 10.1038/nmeth.4612

[28] Polioudakis D , de la Torre-Ubieta L, Langerman J, Elkins AG, Shi X, Stein JL, et al A single-cell transcriptomic atlas of human neocortical development during mid-gestation. Neuron 2019;103:785–801.e8. 31303374 10.1016/j.neuron.2019.06.011 PMC6831089

[29] Johansen N , Somasundaram S, Travaglini KJ, Yanny AM, Shumyatcher M, Casper T, et al Interindividual variation in human cortical cell type abundance and expression. Science 2023;382:eadf2359. 37824649 10.1126/science.adf2359 PMC11702338

[30] Gandal MJ , Zhang P, Hadjimichael E, Walker RL, Chen C, Liu S, et al Transcriptome-wide isoform-level dysregulation in ASD, schizophrenia, and bipolar disorder. Science 2018;362:eaat8127. 30545856 10.1126/science.aat8127 PMC6443102

[31] Saliba AE , Westermann AJ, Gorski SA, Vogel J. Single-cell RNA-seq: advances and future challenges. Nucleic Acids Res 2014;42:8845–60. 25053837 10.1093/nar/gku555 PMC4132710

[32] Yao Z , van Velthoven CTJ, Nguyen TN, Goldy J, Sedeno-Cortes AE, Baftizadeh F, et al A taxonomy of transcriptomic cell types across the isocortex and hippocampal formation. Cell 2021;184:3222–41.e26. 34004146 10.1016/j.cell.2021.04.021 PMC8195859

[33] van der Wijst M , de Vries DH, Groot HE, Trynka G, Hon CC, Bonder MJ, et al The single-cell eQTLGen consortium. Elife 2020;9:e52155. 32149610 10.7554/eLife.52155 PMC7077978

[34] Su C , Xu Z, Shan X, Cai B, Zhao H, Zhang J. Cell-type-specific co-expression inference from single cell RNA-sequencing data. Nat Commun 2023;14:4846. 37563115 10.1038/s41467-023-40503-7 PMC10415381

[35] Lake BB , Chen S, Sos BC, Fan J, Kaeser GE, Yung YC, et al Integrative single-cell analysis of transcriptional and epigenetic states in the human adult brain. Nat Biotechnol 2018;36:70–80. 29227469 10.1038/nbt.4038 PMC5951394

[36] Nagy C , Maitra M, Tanti A, Suderman M, Theroux JF, Davoli MA, et al Single-nucleus transcriptomics of the prefrontal cortex in major depressive disorder implicates oligodendrocyte precursor cells and excitatory neurons. Nat Neurosci 2020;23:771–81. 32341540 10.1038/s41593-020-0621-y

[37] Hodge RD , Bakken TE, Miller JA, Smith KA, Barkan ER, Graybuck LT, et al Conserved cell types with divergent features in human *versus* mouse cortex. Nature 2019;573:61–8. 31435019 10.1038/s41586-019-1506-7 PMC6919571

[38] Bakken TE , Jorstad NL, Hu Q, Lake BB, Tian W, Kalmbach BE, et al Comparative cellular analysis of motor cortex in human, marmoset and mouse. Nature 2021;598:111–9. 34616062 10.1038/s41586-021-03465-8 PMC8494640

[39] Li M , Santpere G, Imamura Kawasawa Y, Evgrafov OV, Gulden FO, Pochareddy S, et al Integrative functional genomic analysis of human brain development and neuropsychiatric risks. Science 2018;362:eaat7615. 10.1126/science.aat7615 PMC6413317 30545854

[40] Braun E , Danan-Gotthold M, Borm LE, Lee KW, Vinsland E, Lönnerberg P, et al Comprehensive cell atlas of the first-trimester developing human brain. Science 2023;382:eadf1226. 37824650 10.1126/science.adf1226

[41] Velmeshev D , Perez Y, Yan Z, Valencia JE, Castaneda-Castellanos DR, Wang L, et al Single-cell analysis of prenatal and postnatal human cortical development. Science 2023;382:eadf0834. 37824647 10.1126/science.adf0834 PMC11005279

[42] Siletti K , Hodge R, Mossi Albiach A, Lee KW, Ding SL, Hu L, et al Transcriptomic diversity of cell types across the adult human brain. Science 2023;382:eadd7046. 37824663 10.1126/science.add7046

[43] Hagai T , Chen X, Miragaia RJ, Rostom R, Gomes T, Kunowska N, et al Gene expression variability across cells and species shapes innate immunity. Nature 2018;563:197–202. 30356220 10.1038/s41586-018-0657-2 PMC6347972

[44] Hao Y , Hao S, Andersen-Nissen E, Mauck WM 3rd, Zheng S, Butler A, et al Integrated analysis of multimodal single-cell data. Cell 2021;184:3573–87.e29. 34062119 10.1016/j.cell.2021.04.048 PMC8238499

[45] Love MI , Huber W, Anders S. Moderated estimation of fold change and dispersion for RNA-seq data with DESeq2. Genome Biol 2014;15:550. 25516281 10.1186/s13059-014-0550-8 PMC4302049

[46] Zappia L , Phipson B, Oshlack A. Splatter: simulation of single-cell RNA sequencing data. Genome Biol 2017;18:174. 28899397 10.1186/s13059-017-1305-0 PMC5596896

[47] Leek JT , Johnson WE, Parker HS, Jaffe AE, Storey JD. The sva package for removing batch effects and other unwanted variation in high-throughput experiments. Bioinformatics 2012;28:882–3. 22257669 10.1093/bioinformatics/bts034 PMC3307112

[48] Robinson MD , McCarthy DJ, Smyth GK. edgeR: a Bioconductor package for differential expression analysis of digital gene expression data. Bioinformatics 2010;26:139–40. 19910308 10.1093/bioinformatics/btp616 PMC2796818

[49] Wang M , Zhao Y, Zhang B. Efficient test and visualization of multi-set intersections. Sci Rep 2015;5:16923. 26603754 10.1038/srep16923 PMC4658477

