# Supplementary material for: Precision and Accuracy in Quantitative Measurement of Gene Expression from Single-cell/nucleus RNA Sequencing Data
Source: Genomics Proteomics Bioinformatics. 2025 Aug 26;23(4):qzaf077. doi: 10.1093/gpbjnl/qzaf077 (PMC12603356; doi:10.1093/gpbjnl/qzaf077)
Supplement: qzaf077_Supplementary_Data [file qzaf077_supplementary_data.zip › Supplementary material captions.docx]

**Supplementary materials**

**Figure S1 Per gene missing rate in pseudo-bulk data with different number of cells**

Missing rate was defined as proportion of zeros in all samples. Data from ROSMAP was used for the demonstration.

Figure S2 Illustration of constructing technical replicates and calculating the CV of expression across technical replicates

**Figure S3 Expression variability at class and subclass level for excitatory neurons**

Data from BICCN_HVS was used. *P* value was calculated with Student’s *t*-test.

**Figure S4 Gene expression variability across technical replicates in mouse brain snRNA-seq data**

**A.** Median CV across genes in replicates constructed at class and subclass level. **B.** Linear regression model of cell numbers in replicates and median CV identified at class and subclass level.

**Figure S5 Reduction of CV with the increased number of cells sequenced in three non-brain tissues**

Data from Tabula Sapiens consortium was used. Red line denotes CV of 0.1.

**Figure S6 Expression abundance and CV in ROSMAP data**

**A.** Relationship of expression and CV in excitatory neuron from ROSMAP study. **B.** CV of marker genes. Blue and red line denote CV of 0.2 and 0.1. CPM, counts per million.

**Figure S7 Expression variability in scRNA-seq and snRNA-seq data**

Three human microglia samples with both sc/snRNA-seq data are shown.

**Figure S8 Proportions of samples with acceptable expression variability**

**A.** The average and maximum percentages of samples that satisfy the precision criterion. **B.** and **C.** Examples from BICCN_adult (B) and BICCN_HVS (C) datasets were used for illustration. Samples achieving the precision threshold, defined by a CV of 0.1 or lower, are indicated in green, signifying acceptable expression precision, while those failing to meet the threshold are marked in yellow, indicating low precision. Instances where a cell type is not represented in a sample are left blank. The accompanying bar plot provides a detailed breakdown of the exact proportion of samples that satisfy the precision criterion.

**Figure S9 Expression variability across replicates in human microglia**

**Figure S10 Association between technical factors and gene expression variability across technical replicates in snRNA-seq data**

**A.** Median CV across detected genes in replicates versus total sequencing depth. **B.** Median CV across detected genes in replicates versus total sequencing saturation rate. **C.** Comparison of median CV across detected genes in replicates between data sequenced by 10X Chromium and Smart-seq platforms.

**Figure S11 Relationship between expression accuracy and the number of cells in simulated data**

The X-axis represents the number of cells in each sample, and the Y-axis shows the percentage of genes with good accuracy, as defined by Pearson correlation and a linear regression model.

**Figure S12 True positive rate of DEGs with different expression levels categorized by log-transformed CPM**

**Figure S13 Applying the 500-cell threshold and SNR to schizophrenia case–control scRNA-seq data (Ruzicka et al)**

**A.** Impact of cell number cutoff on the reproducibility of DEGs in two schizophrenia cohorts. The plot illustrates the effect of different cell number cutoffs on the reproducibility of DEGs identified in two independent schizophrenia cohorts (MCL and Mt Sinai). **B.** The relationship between SNR and DEG reproducibility in astrocytes.

**Table S1 Expression accuracy statistics in datasets from four species**
