# Supplementary figures and images for: Precision and Accuracy in Quantitative Measurement of Gene Expression from Single-cell/nucleus RNA Sequencing Data
Source: Genomics Proteomics Bioinformatics. 2025 Aug 26;23(4):qzaf077. doi: 10.1093/gpbjnl/qzaf077 (PMC12603356; doi:10.1093/gpbjnl/qzaf077)

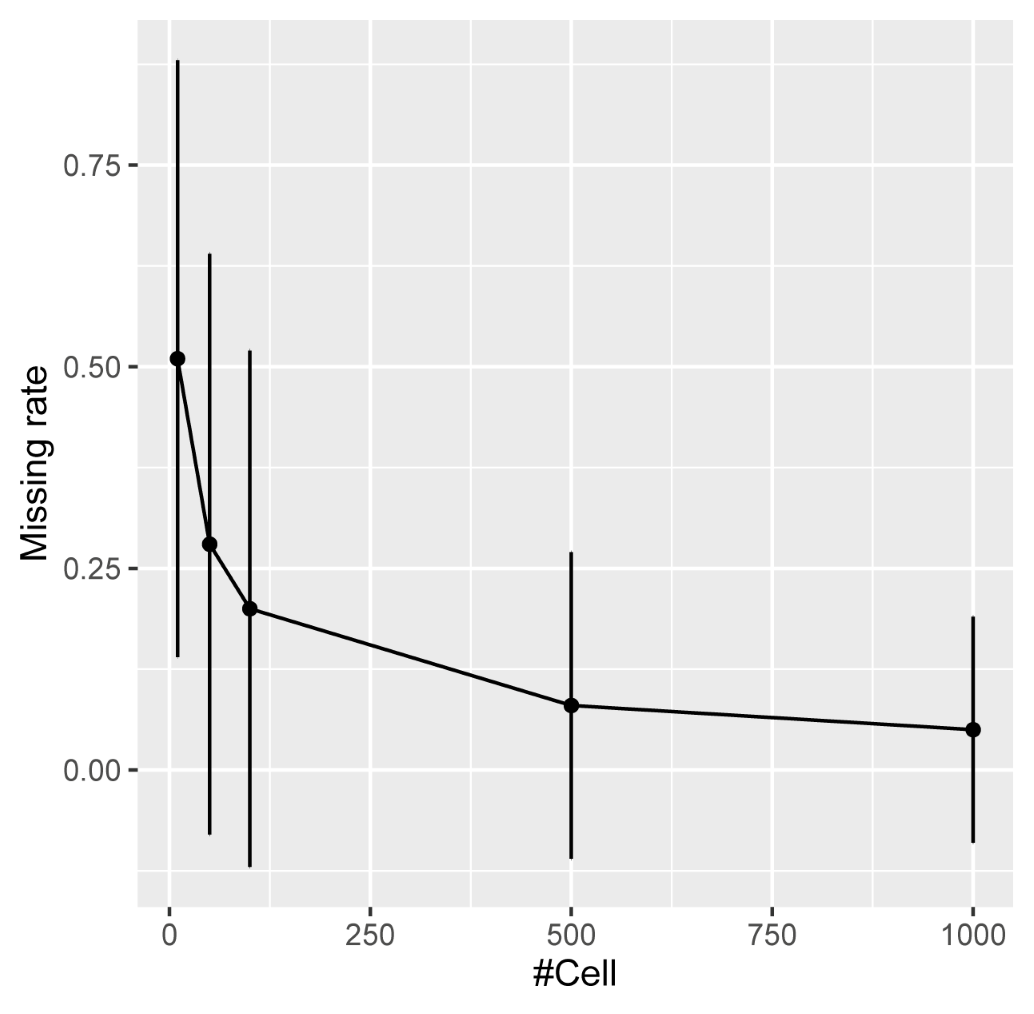

Supplement: qzaf077_Supplementary_Data [file qzaf077_supplementary_data.zip › Figure S1.tif]

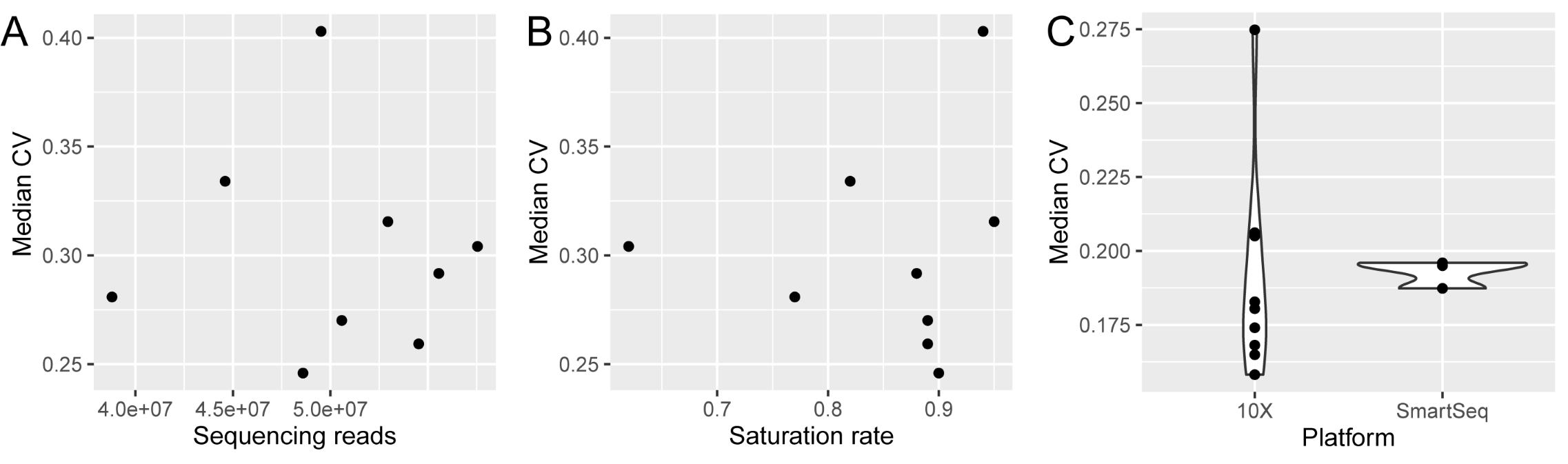

Supplement: qzaf077_Supplementary_Data [file qzaf077_supplementary_data.zip › Figure S10.tif]

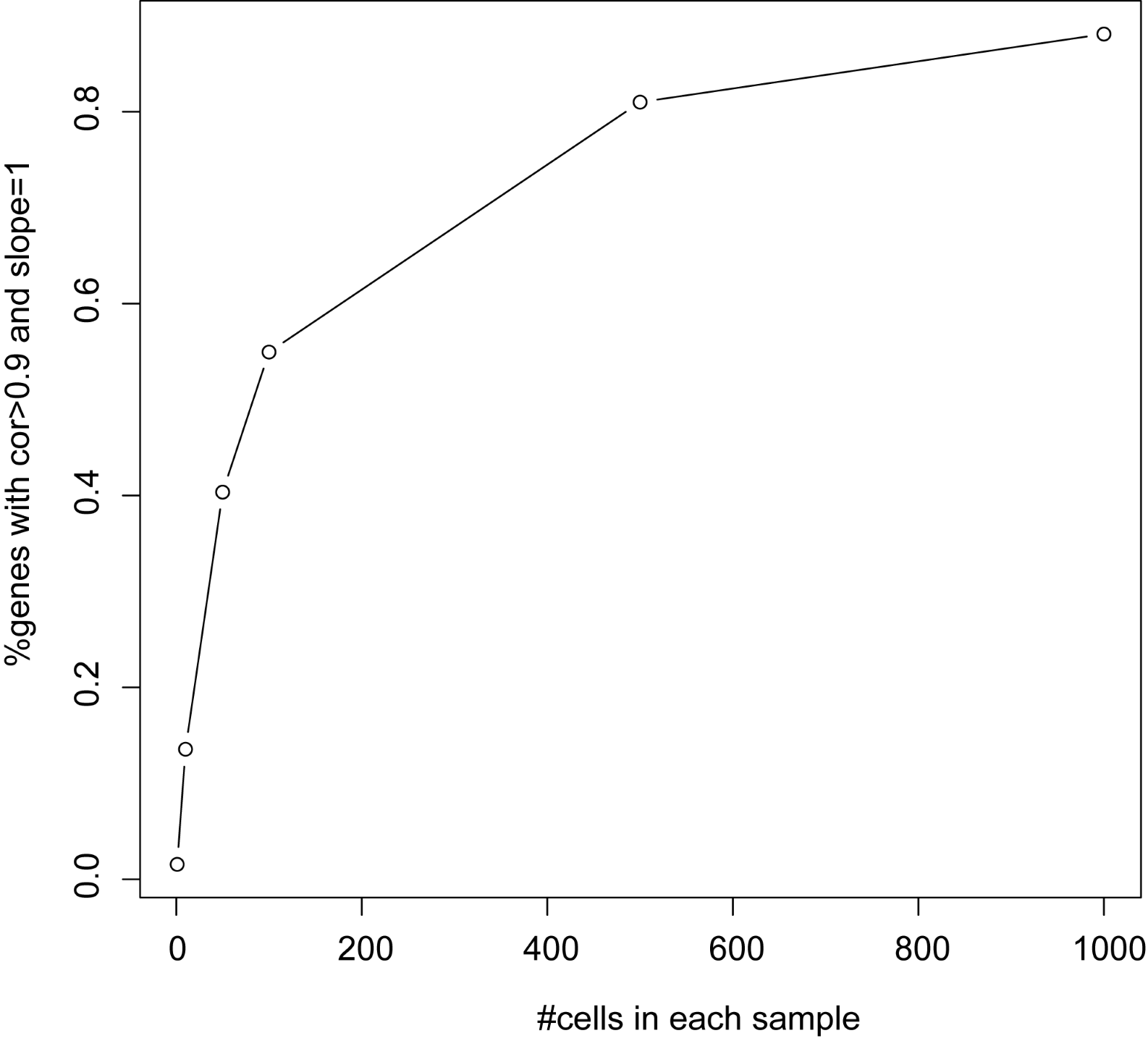

Supplement: qzaf077_Supplementary_Data [file qzaf077_supplementary_data.zip › Figure S11.tif]

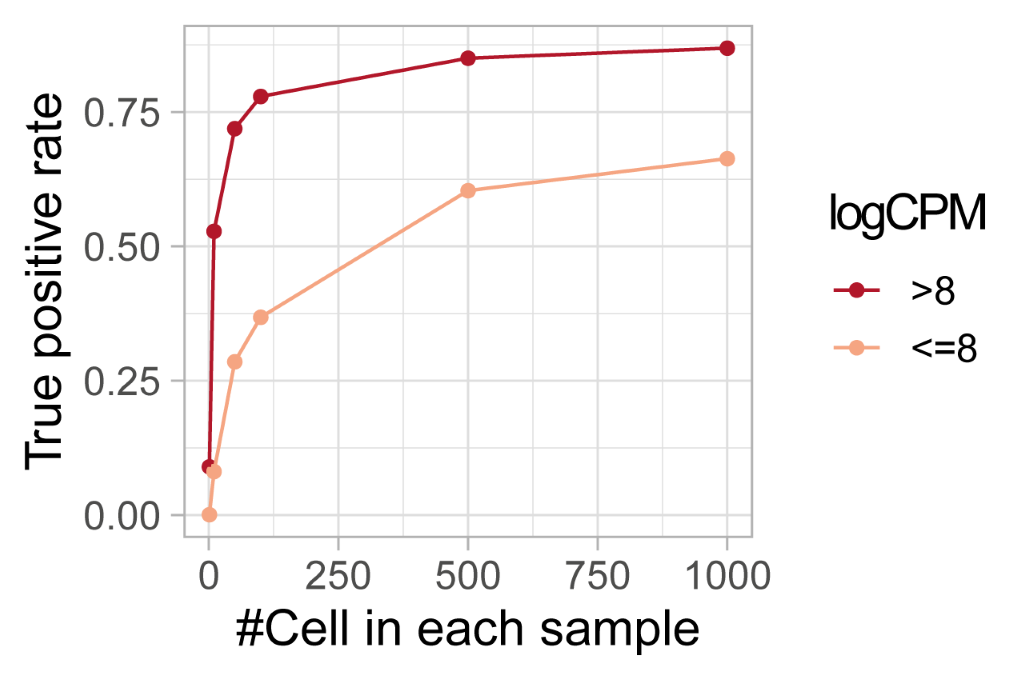

Supplement: qzaf077_Supplementary_Data [file qzaf077_supplementary_data.zip › Figure S12.tif]

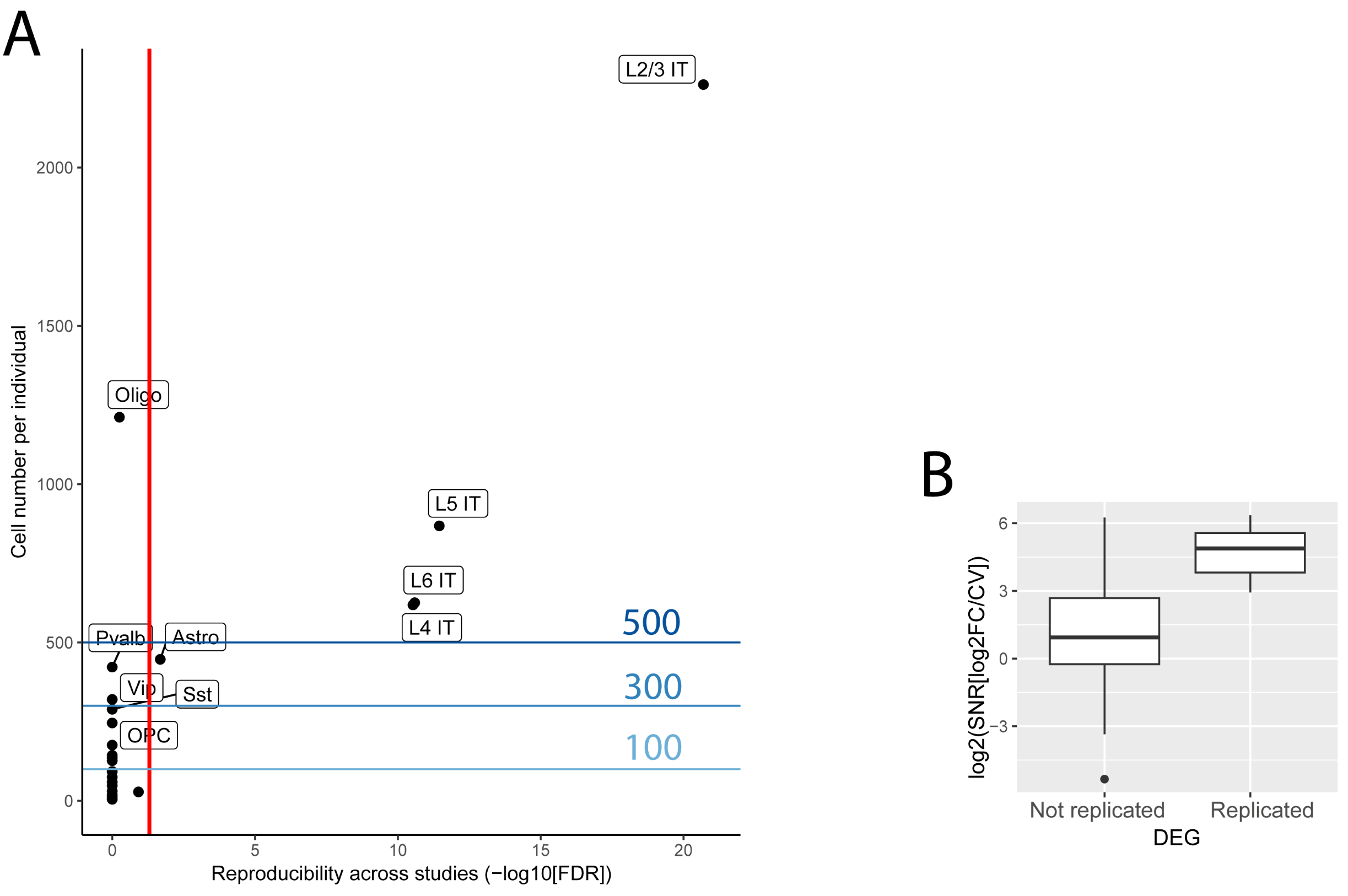

Supplement: qzaf077_Supplementary_Data [file qzaf077_supplementary_data.zip › Figure S13.tif]

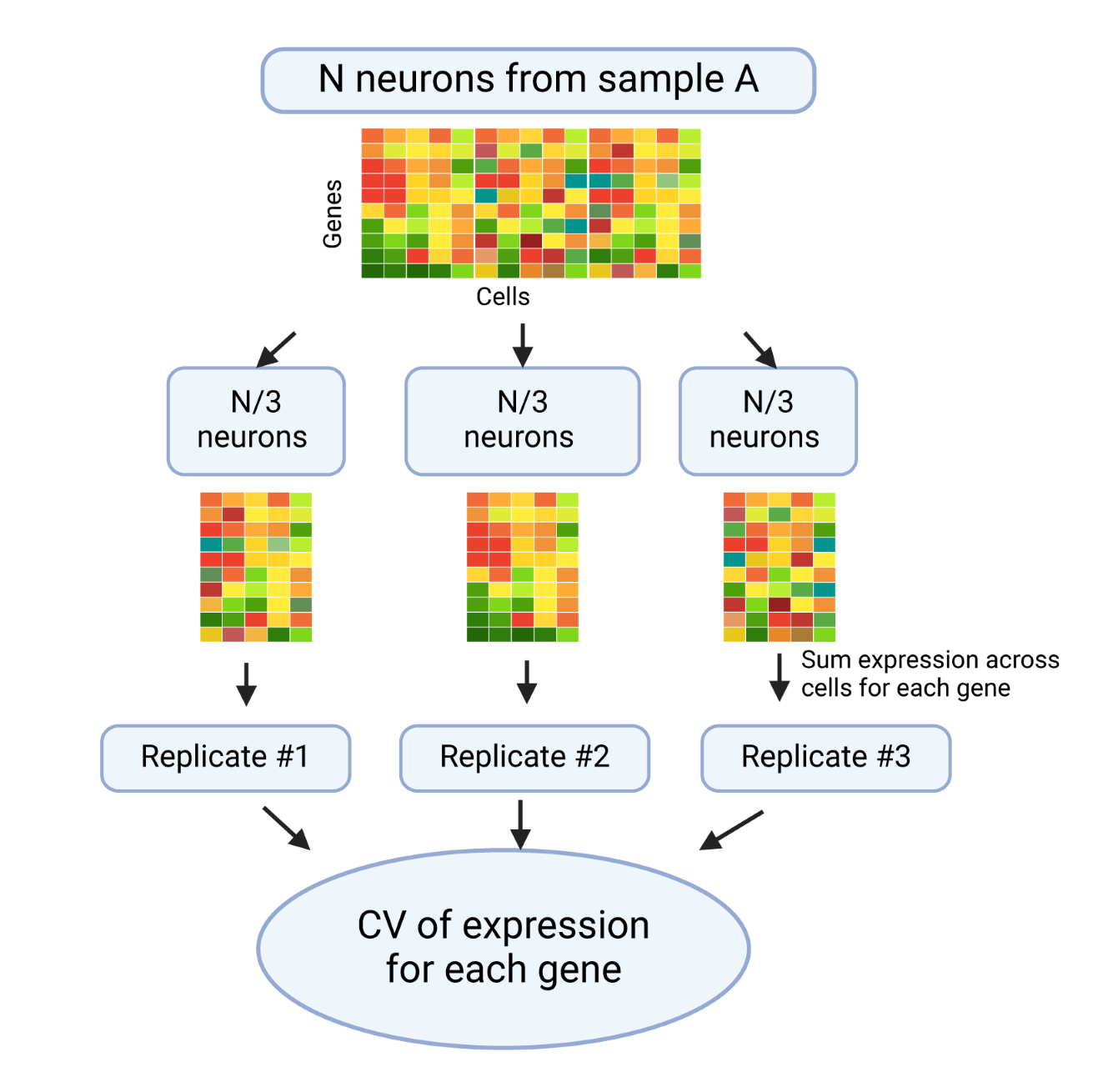

Supplement: qzaf077_Supplementary_Data [file qzaf077_supplementary_data.zip › Figure S2.tif]

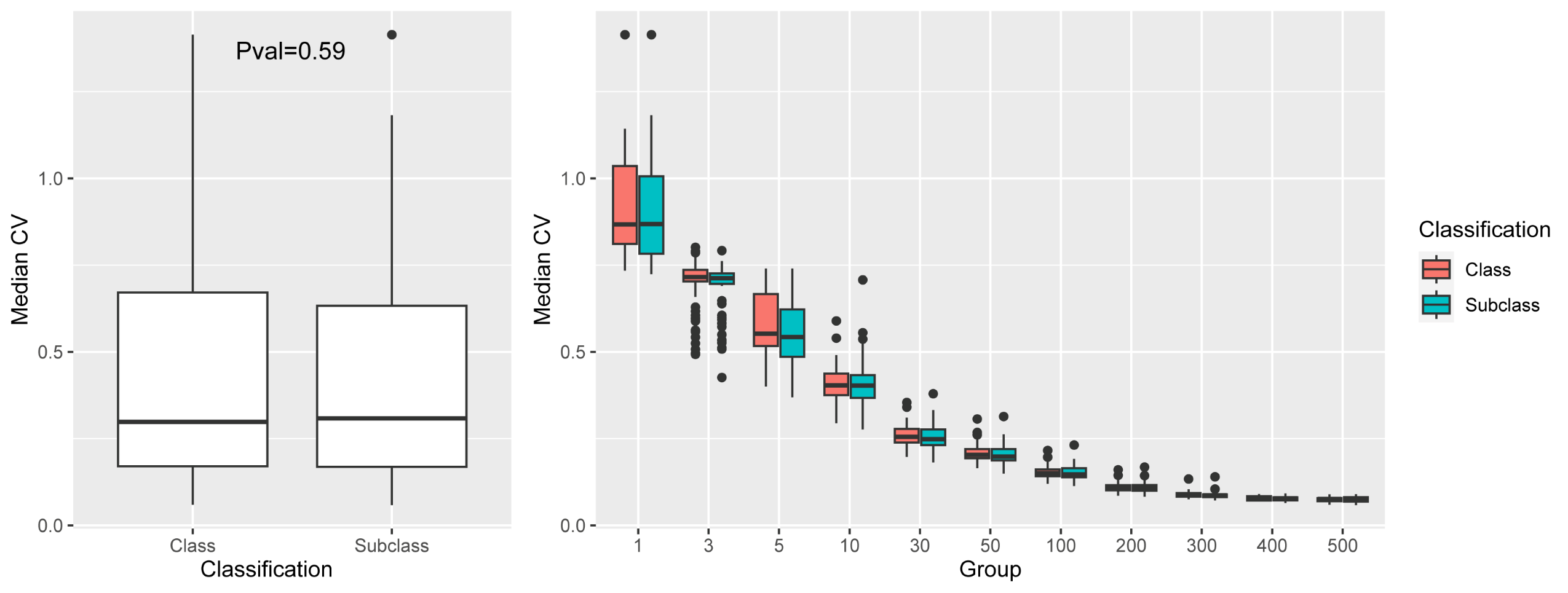

Supplement: qzaf077_Supplementary_Data [file qzaf077_supplementary_data.zip › Figure S3.tif]

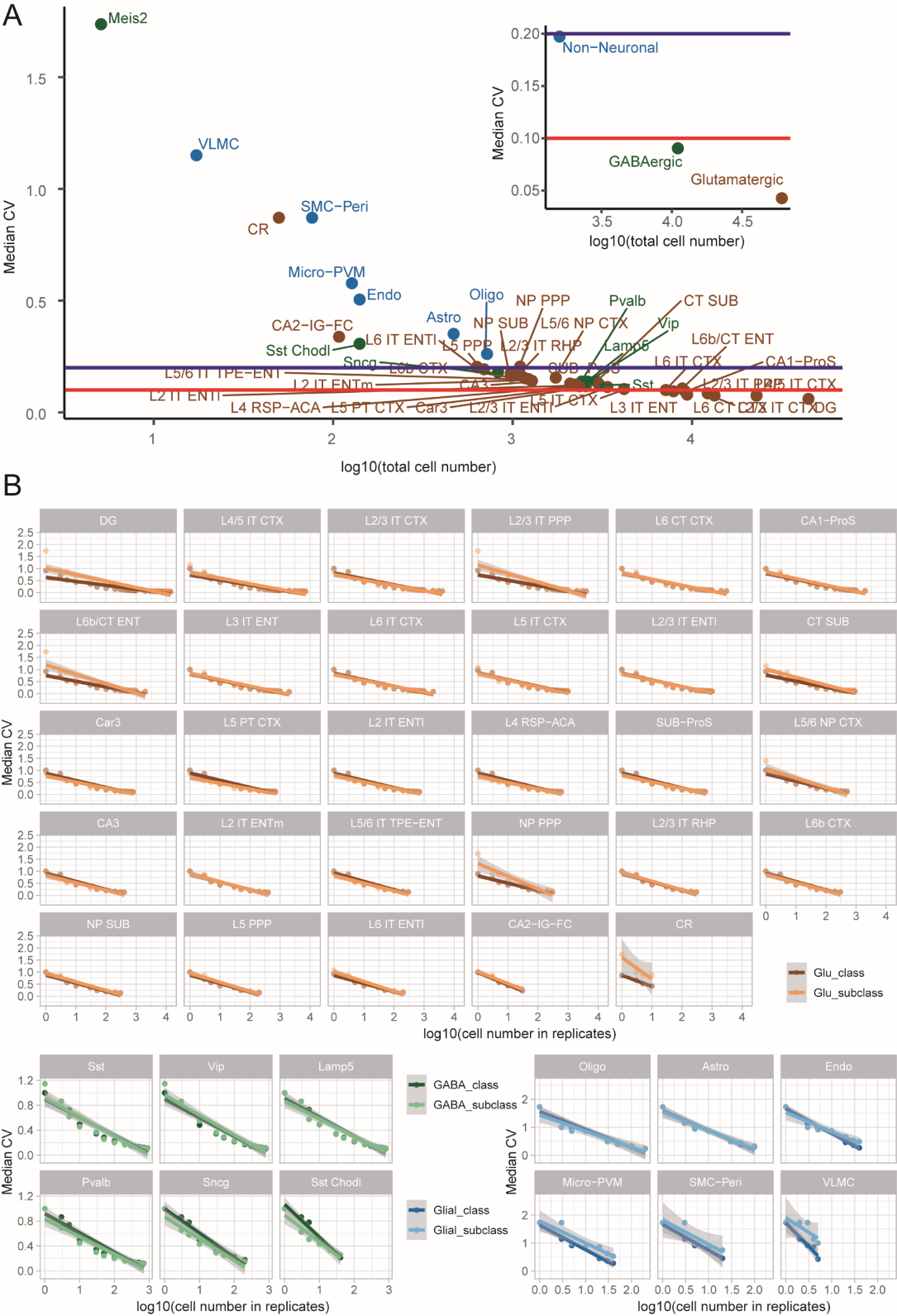

Supplement: qzaf077_Supplementary_Data [file qzaf077_supplementary_data.zip › Figure S4.tif]

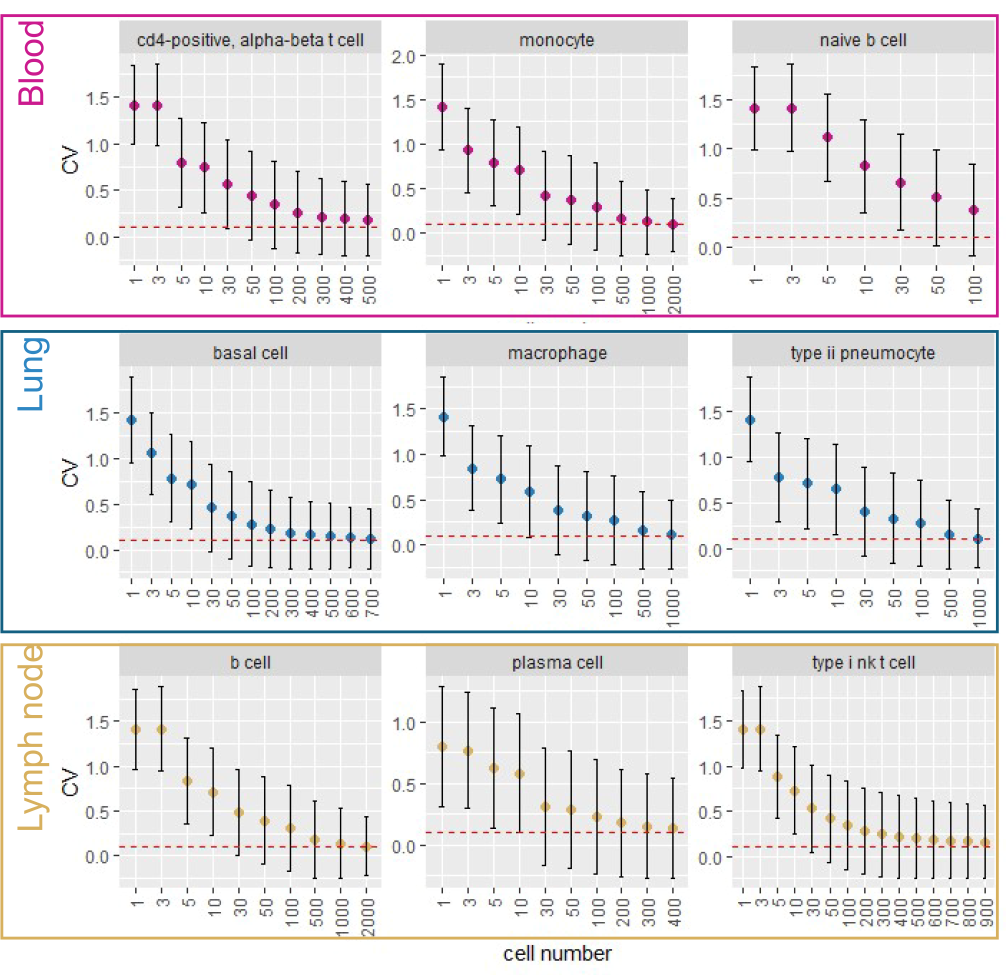

Supplement: qzaf077_Supplementary_Data [file qzaf077_supplementary_data.zip › Figure S5.tif]

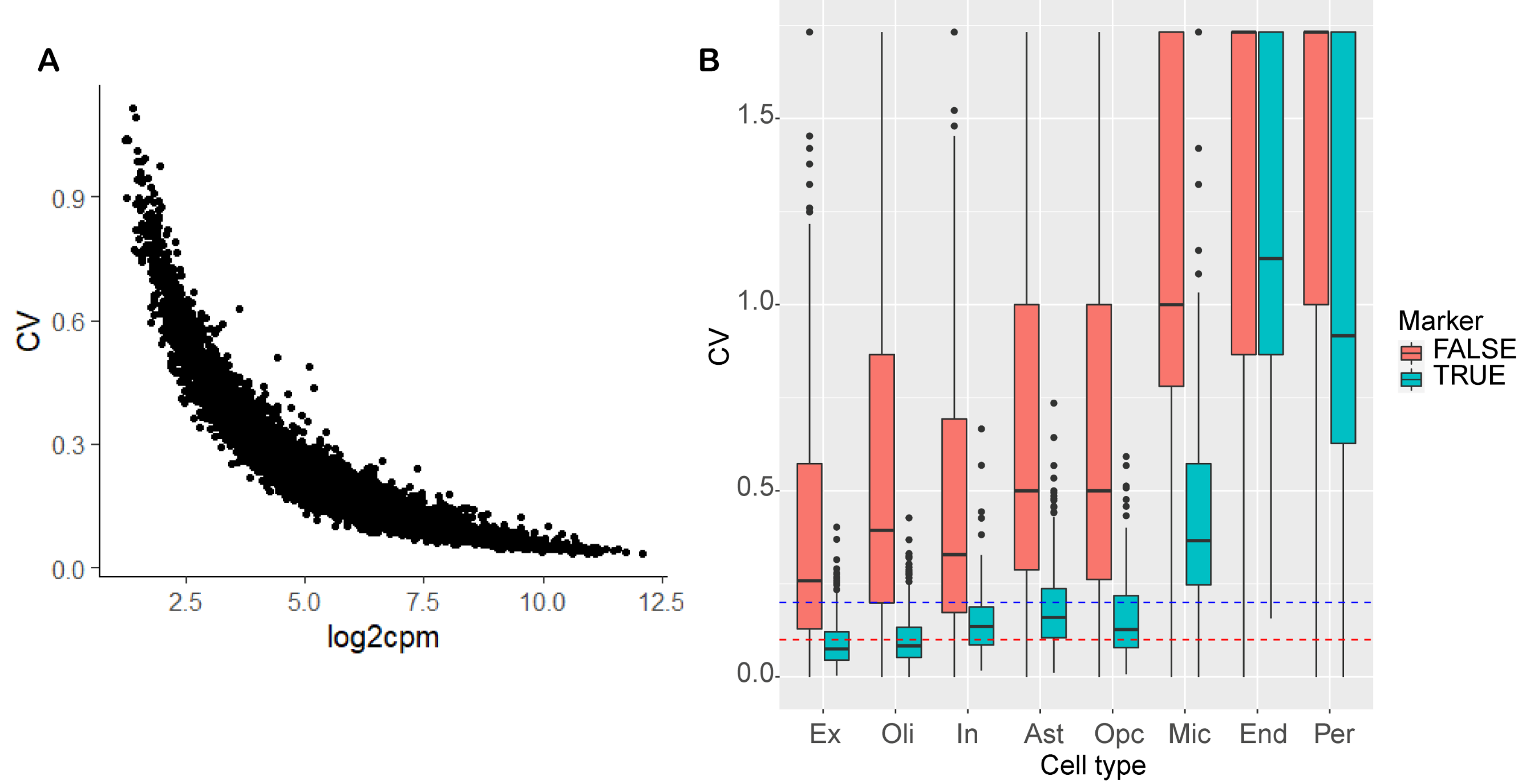

Supplement: qzaf077_Supplementary_Data [file qzaf077_supplementary_data.zip › Figure S6.tif]

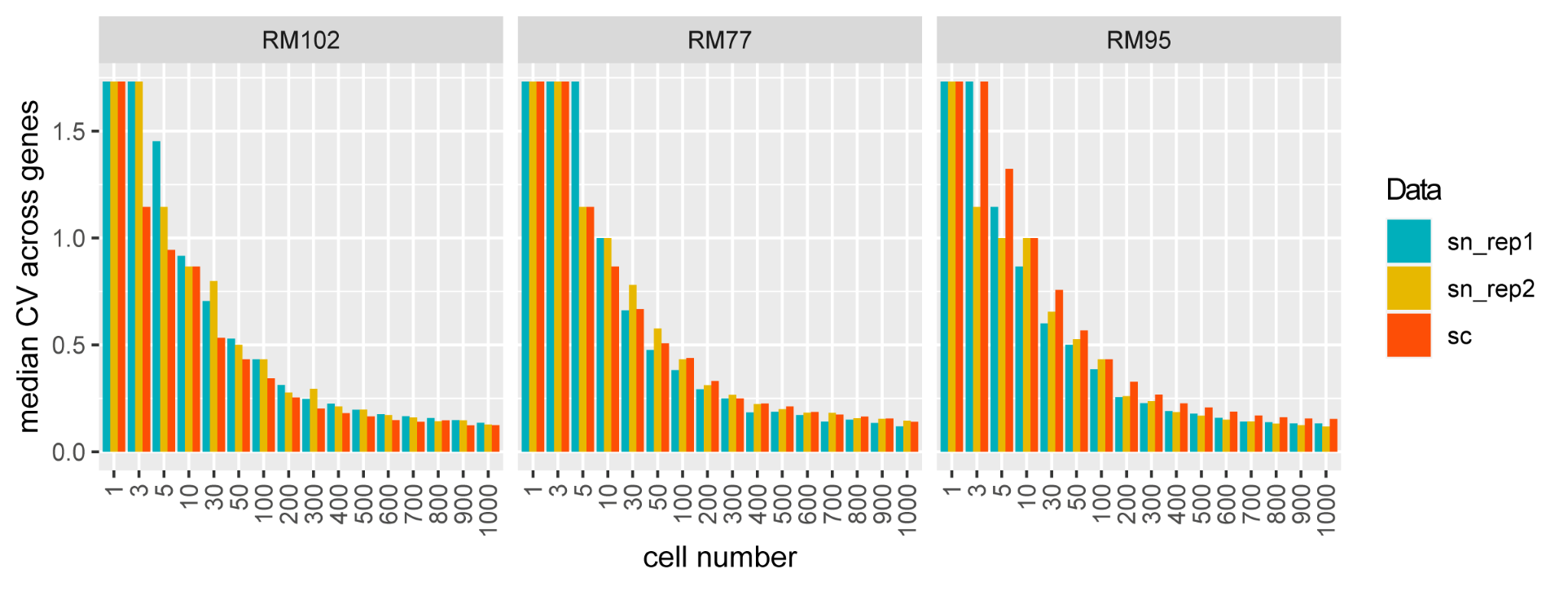

Supplement: qzaf077_Supplementary_Data [file qzaf077_supplementary_data.zip › Figure S7.tif]

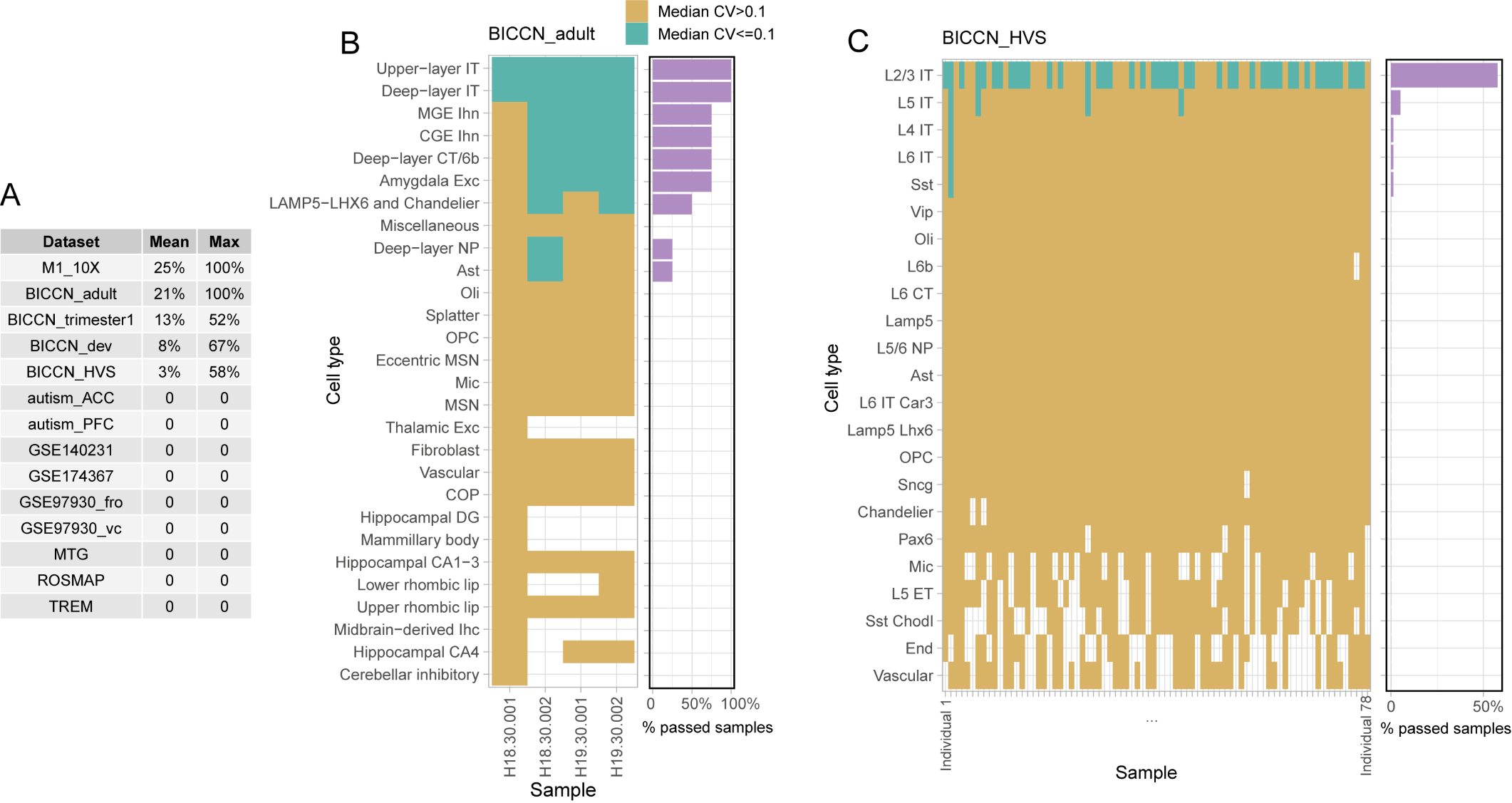

Supplement: qzaf077_Supplementary_Data [file qzaf077_supplementary_data.zip › Figure S8.tif]

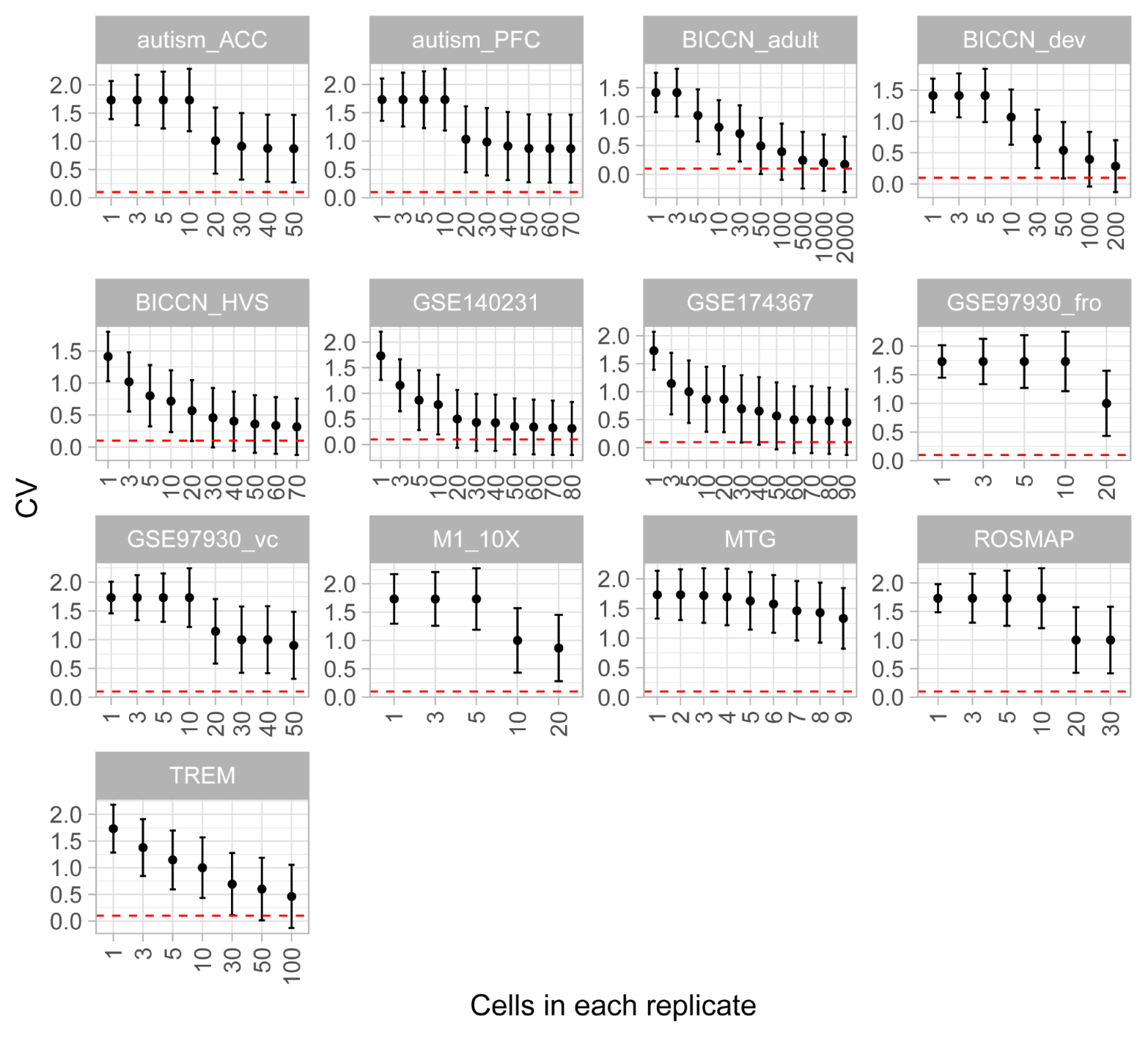

Supplement: qzaf077_Supplementary_Data [file qzaf077_supplementary_data.zip › Figure S9.tif]
